# (Pan)genomic analysis of two *Rhodococcus* isolates and their role in phenolic compound degradation

**DOI:** 10.1128/spectrum.03783-23

**Published:** 2024-02-20

**Authors:** Sarah Benning, Karin Pritsch, Viviane Radl, Roberto Siani, Zhongjie Wang, Michael Schloter

**Affiliations:** 1 Research Unit for Comparative Microbiome Analysis, Helmholtz Munich, German Research Center for Environmental Health, Neuherberg, Germany; 2 Research Unit for Environmental Simulations, Helmholtz Munich, German Research Center for Environmental Health, Neuherberg, Germany; 3 Chair for Environmental Microbiology, TUM School of Life Sciences, Technical University Munich, Munich, Germany; Dominican University New York, Orangeburg, South Carolina, USA

**Keywords:** actinobacteria, comparative genomics, biphenyl degradation, benzoate degradation, apple replant disease

## Abstract

The genus *Rhodococcus* is recognized for its potential to degrade a large range of aromatic substances, including plant-derived phenolic compounds. We used comparative genomics in the context of the broader *Rhodococcus* pan-genome to study genomic traits of two newly described *Rhodococcus* strains (type-strain *Rhodococcus pseudokoreensis* R79^T^ and *Rhodococcus koreensis* R85) isolated from apple rhizosphere. Of particular interest was their ability to degrade phenolic compounds as part of an integrated approach to treat apple replant disease (ARD) syndrome. The pan-genome of the genus *Rhodococcus* based on 109 high-quality genomes was open with a small core (1.3%) consisting of genes assigned to basic cell functioning. The range of genome sizes in *Rhodococcus* was high, from 3.7 to 10.9 Mbp. Genomes from host-associated strains were generally smaller compared to environmental isolates which were characterized by exceptionally large genome sizes. Due to large genomic differences, we propose the reclassification of distinct groups of rhodococci like the *Rhodococcus equi* cluster to new genera. Taxonomic species affiliation was the most important factor in predicting genetic content and clustering of the genomes. Additionally, we found genes that discriminated between the strains based on habitat. All members of the genus *Rhodococcus* had at least one gene involved in the pathway for the degradation of benzoate, while biphenyl degradation was mainly restricted to strains in close phylogenetic relationships with our isolates. The ~40% of genes still unclassified in larger *Rhodococcus* genomes, particularly those of environmental isolates, need more research to explore the metabolic potential of this genus.

**IMPORTANCE:**

*Rhodococcus* is a diverse, metabolically powerful genus, with high potential to adapt to different habitats due to the linear plasmids and large genome sizes. The analysis of its pan-genome allowed us to separate host-associated from environmental strains, supporting taxonomic reclassification. It was shown which genes contribute to the differentiation of the genomes based on habitat, which can possibly be used for targeted isolation and screening for desired traits. With respect to apple replant disease (ARD), our isolates showed genome traits that suggest potential for application in reducing plant-derived phenolic substances in soil, which makes them good candidates for further testing against ARD.

## INTRODUCTION

Members of the genus *Rhodococcus* are found in various environments, including soil, water, and plants (1), as well as in association with various hosts (2). With a few exceptions of animal, human (3, 4), and plant pathogens (5), the majority of *Rhodococcus* members are considered nonpathogenic. *Rhodococcus* species are known for their remarkable metabolic capabilities and adaptive nature (6, 7). Besides their abilities to produce antibiotic substances which are frequently used in medicine, and other valuable compounds for biotechnology (8), one of the most notable characteristics of the *Rhodococcus* genus is their ability to degrade a wide range of natural and synthetic organic compounds (9, 10). This includes hydrocarbons like alkanes, aromatic, and polycyclic aromatic hydrocarbons (11–15), as well as steroids (16). These high metabolic capacities are often associated with large and linear chromosomes (17), with usually linear plasmids and megaplasmids (7). The linear plasmids most likely play a role in horizontal gene transfer of genes coding for degradation of aromatic compounds, especially within the genus *Rhodococcus* itself (18). The genus *Rhodococcus* was first described by Zopf in 1891 (19). It consists of Gram-positive, aerobic, nonsporulating Actinobacteria. There was and is ongoing taxonomic (re-)classification based on molecular methods (20, 21), as many members of the genus had been included before the use of a polyphasic taxonomic approach (22). Due to large differences of species within the genus, there are considerations to split *Rhodococcus* into more than one genus (21, 23).

Apple roots are known to produce and exude phenolic compounds, which are naturally occurring defense compounds like phytoalexins, but also hypothesized to contribute to the development of apple replant disease (ARD), a syndrome that prevents sustainable apple cultivation (24). A metagenomics-based study by Radl et al. in 2019 (25) suggested a reduced potential for degradation of phenolic compounds, especially benzoate, to be related to a lower relative abundance of certain actinobacterial genera in ARD compared to control soil. Subsequently, we isolated two *Rhodococcus* strains from the rhizosphere of healthy apple plants [*Rhodococcus pseudokoreensis* R79^T^ and *Rhodococcus koreensis* R85 (26, 27)], which showed high potential for the degradation of phytoalexins. In the frame of this study, we integrated the two new genomes into the greater *Rhodococcus* pan-genome. Using comparative genome analysis, we wanted to provide information on the genetic variability of*,* as well as the evolutionary relationships within the genus. As it is known that habitat can have a significant impact on the genetic diversification in microbes, like for the closely related *Streptomyces* (28), we hypothesized that not only species affiliation alone would determine the genetic similarity of the genomes, but that it would also be significantly influenced by habitat/isolation source of the strains (H1). Furthermore, we screened for genes encoding the degradation of phenolic compounds in our isolates, as we hypothesized that phenol degradation might be an important trait for coping with the release of aromatic compounds in the rhizosphere of apple (H2). By investigating how the genes for degradation of phenolics like benzoate and biphenyl are distributed across the genus we elucidated if also other members in the genus *Rhodococcus* could be possible candidates for remediating apple replant disease.

## RESULTS

### General features of the *Rhodococcus* pan-genome and phylogenetic analysis

**Fig 1:**
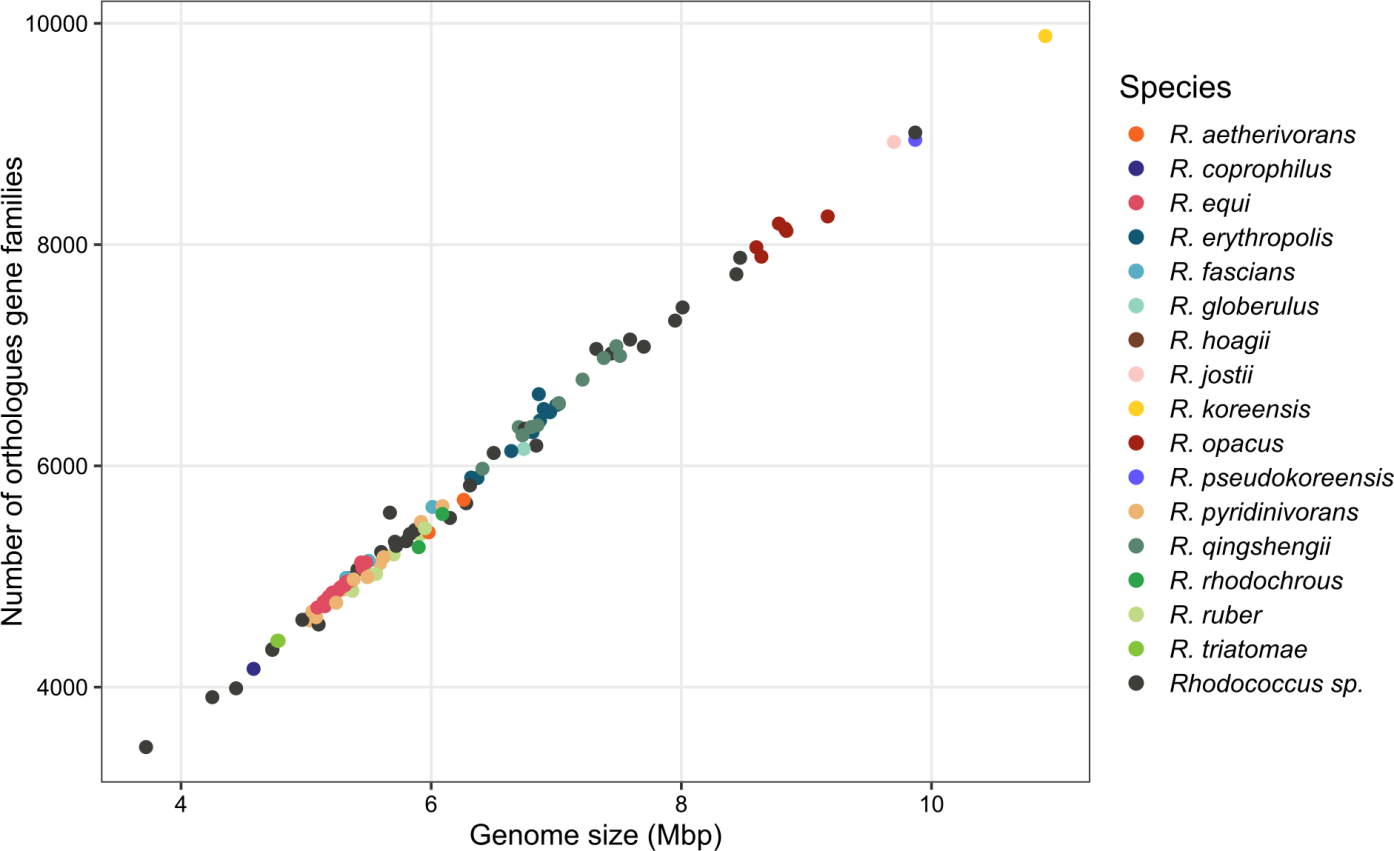
Genome size vs number of orthologues gene families per strain, colored by taxonomy. Members of the same species are grouped together, still, there is a range of ~1 Mbp length variation also within a species. *R. koreensis* had by far the largest genome, followed by *R. pseudokoreensis* and closely related species.

**Fig 2:**
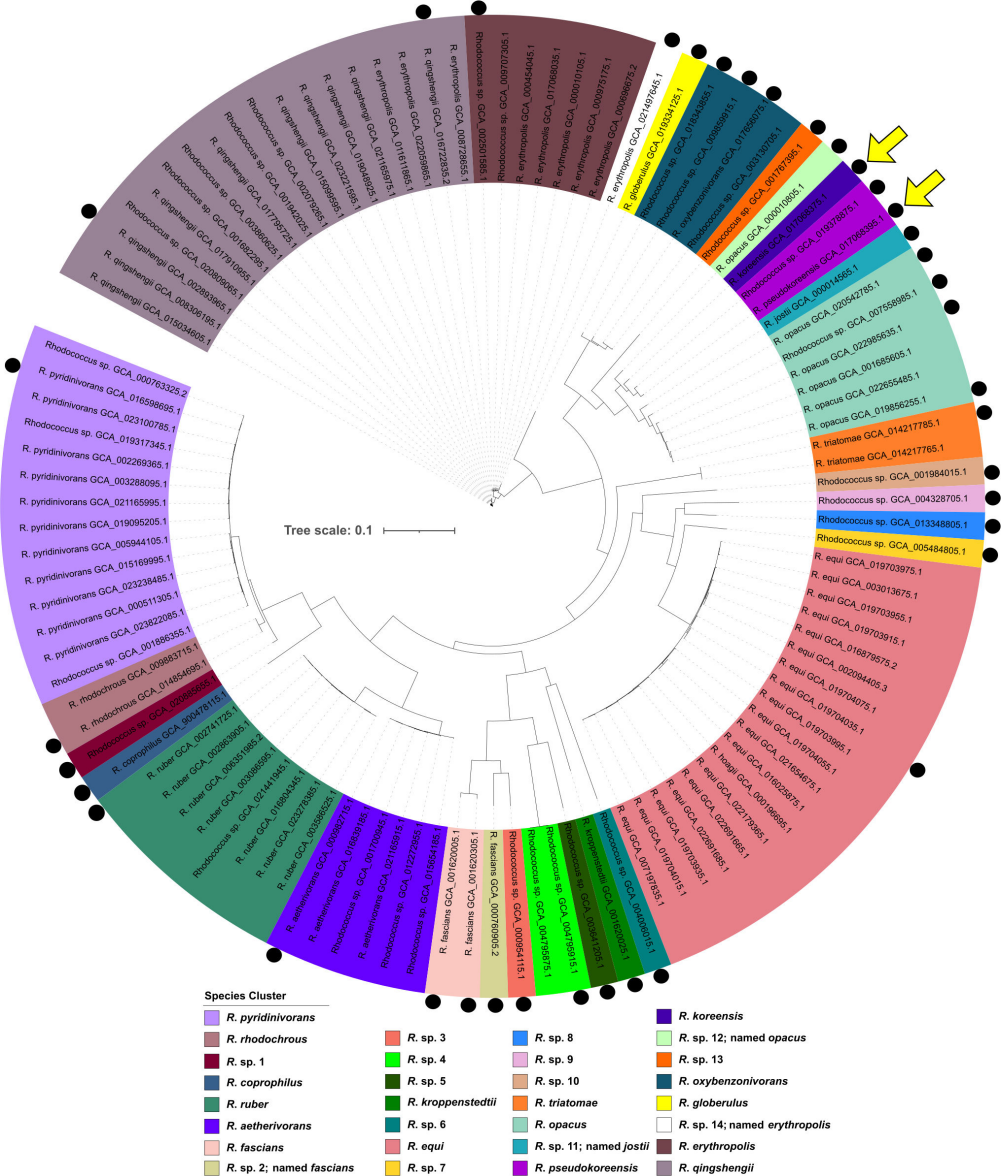
Phylogenetic tree of all 109 selected genomes calculated from the core genome alignment, displaying 30 species-specific clusters with dDDH > 70%. The 38 medoid genomes used in the downstream analysis are marked with a black circle. Yellow arrows indicate the isolates of this study. Box colors are respective species clusters.

The pan-genome of 109 complete, high-quality *Rhodococcus* genomes contained 83,399 genes in total, with only 1,085 core genes (core + soft core, genes present in >95% = 107 strains), which represented 1.3% of the total pan-genome. All core genes were assigned to basic cell functioning. Out of the total, 9,475 genes (11.4%) were shell genes and 72,839 (87.3%) were accessory genes. Features of the pan-genome of the 109 strains can be found in Fig. S1 to S4. Regarding the size of the genomes, clusters with clearly species-specific genome sizes could be detected (Fig. 1), albeit with a range of variation up to 1 Mbp within a species. Commonly pathogenic *Rhodococcus equi* strains showed a relatively small variation in genome size compared to other *Rhodococcus* species. In general, strains with a host-associated lifestyle showed smaller genome sizes with an average of 5.3 Mbp (std 0.6 Mbp) than free-living *Rhodococcus* strains with an average of 6.6 Mbp (std 1.3 Mbp) (Fig. S5). The smallest genome (*Rhodococcus* sp. GCA_004006015.1, isolated from an antelope) with 3.72 Mbp was only 34% of the size of the largest genome (*R. koreensis*). Furthermore, their digital DNA–DNA hybridization (dDDH) had a 19.8% similarity and a GC (guanine-cytosine) content difference of 5.02%. Genomes of strains originating from soil and sediments were in the range from 4.58 Mbp (*Rhodococcus coprophilus* GCA_900478115.1—lake sediment) to 10.91 Mbp (*R. koreensis* GCA_017068375.1—rhizosphere). Genome size was almost directly translatable to the number of orthologous gene families (Fig. 1). Interestingly, isolate *R. koreensis* R85 had the largest genome of all 109 strains, followed by *Rhodococcus* sp. GCA_019378875.1, *R. pseudokoreensis* R79^T^, and the closely related *Rhodococcus jostii* GCA_000014565.1; all these strains were derived from soil or sediment. The 109 strains were assigned to 30 species-specific clusters with dDDH values > 70% (Fig. 2). Half of the strains denoted as *Rhodococcus* sp. were distinctly placed within clusters of known species. Some of the *Rhodococcus erythropolis* strains were assigned to the *Rhodococcus qingshengii* cluster. However, 11 *Rhodococcus* sp. strains could not be assigned to any known species and build 10 clusters of potentially new species. Similarly, four genomes previously assigned to already described species are potentially new species. *R. erythropolis* GCA_021497645.1, *Rhodococcus opacus* GCA_000010805.1, and *Rhodococcus fascians* GCA_000760905 had dDDH values of less than 55%, 43%, and 27%, respectively, compared to other species members. *R. jostii* GCA_000014565.1 had dDDH values of 41.4% and 41.3% compared to *R. jostii* DSM 44719 and *R. jostii* NBRC 16295, respectively, although GC-content was similar with differences of 0.1% and 0.07%. All suggested new taxonomic affiliations of this study can be found in Table S1. The isolation source was not correlated with the taxonomic affiliation of the strains (Fig. S6). Genome comparison of *R. pseudokoreensis* R79^T^ and *Rhodococcus* sp. GCA_019378875.1 revealed a dDDH value of 70.2%, a GC difference of 0.08%, and an Average Nucleotide Identity (ANI) value of 96.1 suggesting *Rhodococcus* sp. GCA_019378875.1 is probably another strain belonging to the species *R. pseudokoreensis*.

**Fig 3:**
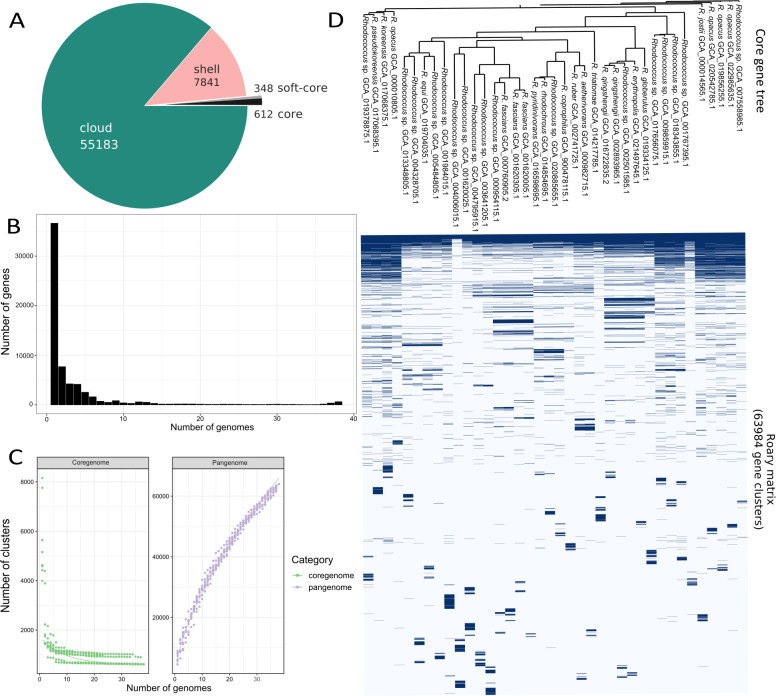
Features of the pan-genome for the 38 medoid *Rhodococcus* genomes. All graphs are based on the gene presence-absence matrix obtained with roary v3.13.0. (A) Pan-genome-pie. A number of core/soft core, shell, and cloud (accessory) gene clusters that are present in 99%/95%, 15%, or only in one or very few genomes, respectively. (B) Frequency barplot of gene cluster vs number of genomes. It is indicated, how many genes are present in one genome, two genomes… to all 38 genomes, which represent the core. The number of genes unique to one or only several strains is very high, the core is reduced to basic cell functioning. (C) Pan-genome and core genome curves. The pan-genome still shows features of an open pan-genome. (D) Gene presence/absence binmap showing a cluster of orthologous genes per genome.

**Fig 4:**
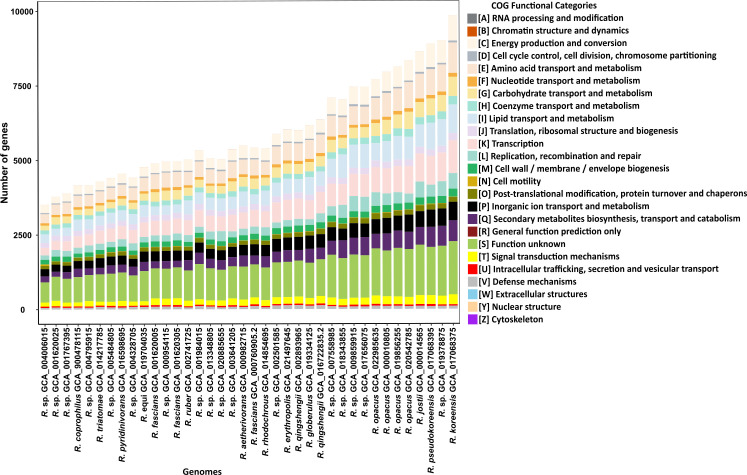
COG functional categories of 38 medoid genomes, annotated with Prodigal v2.6.3. Functional annotation was done using eggnogg-mapper v2.1.9 and the EggNOG database v5.0 orthology assignments. Strains were sorted by ascending genome size. The genus name *Rhodococcus* is always abbreviated as *R*.

The genome data set was balanced using symmetric pairwise ANI dissimilarities (Fig. S7). This resulted in 38 medoid *Rhodococcus* genomes (the most centrally located genomes of each similarity cluster) for further analysis. Every species-specific cluster was represented by at least one medoid genome (Fig. 2). The pan-genome of the 38 medoid genomes had 63,984 gene clusters in total. Among them, 1.5% of the genes were core genes (core + soft core, genes in >36 = 95% of strains), 12.3% shell genes and 86.2% accessory genes present only in less than 5 = 15% of strains (Fig. 3A). This distribution was highly similar to the pan-genome of the 109 strains (Fig. S1 to S4). Most gene clusters were only present in one single strain (Fig. 3B). As more genomes were added to the pan-genome, the total number of gene clusters increased, while the number of core genes tended to be consistent, indicating an open pan-genome (Fig. 3C). Figure 3D shows the gene clusters in relation to the phylogenetic tree. The large variation in the accessory genome is visible, as well as the small core, consisting of genes assigned to basic cell functioning. In general, genes of the pan-genome could be assigned to 25 functional clusters of orthologous genes (COG)–categories (Fig. 4). The ratio of COG categories within each strain was similar for all, with genes coding for the COG categories “Transcription,” “Lipid transport and metabolism,” and “Amino acid transport and metabolism” being the largest assigned categories. However, the largest category in all genomes was genes of unknown function, with an average of 20.12% per genome.

### Functional analysis

**Fig 5:**
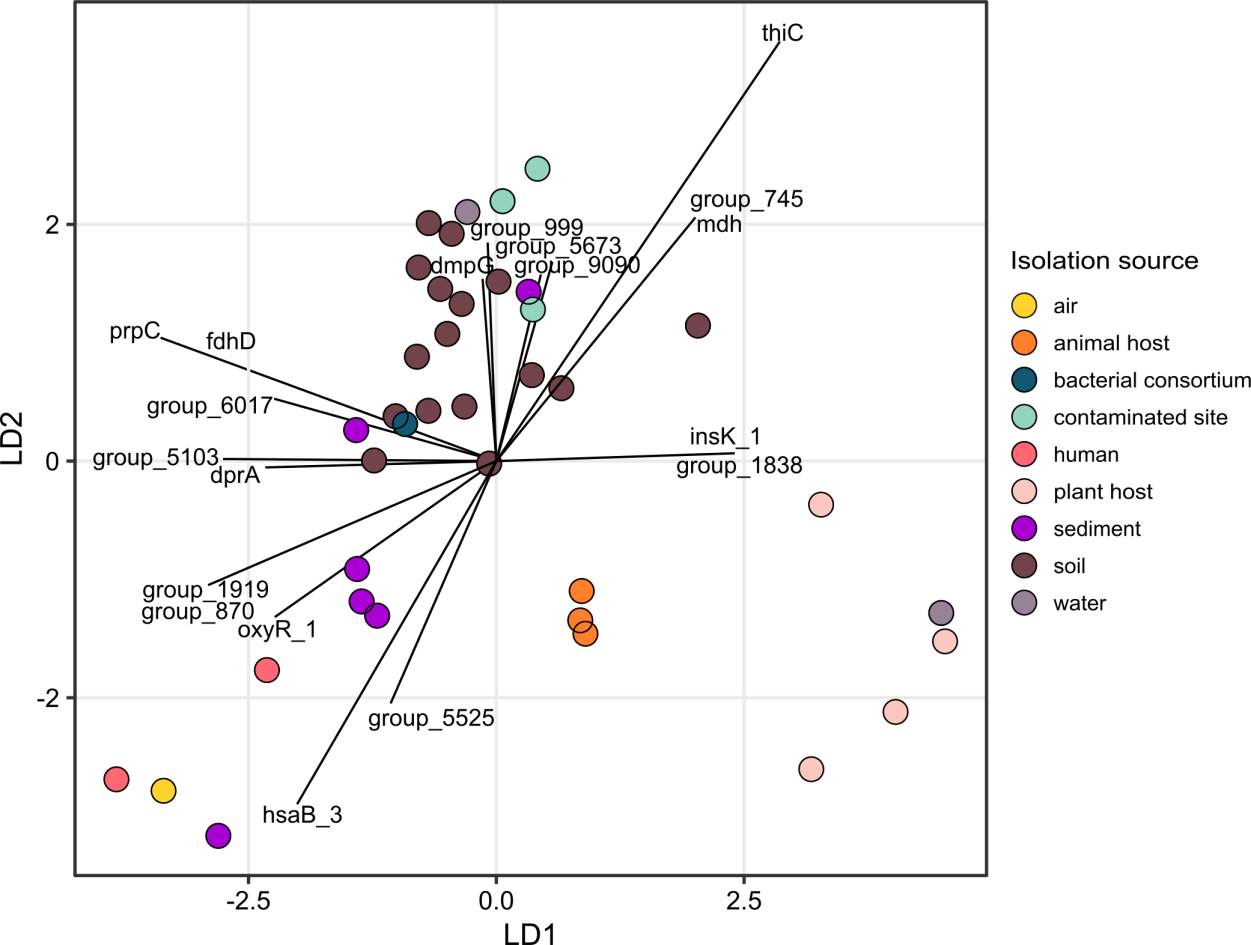
LDA plot of genes of 38 medoid genomes important for the discrimination of isolation source of the strains. Model response variable: isolation source. Only genes of the 99% quantile are shown.

Based on Principle Component Analysis (PCA) the genomes showed a clear taxonomic clustering (data not shown). In addition, linear discriminant analysis (LDA) resulted in separating the genomes according to their isolation source (Fig. 5). The LDA clustering showed a clear separation between plant, animal and human host strains. Strains coming from soil could be clearly distinguished from sediment samples. Samples from contaminated sites clustered further together on top of the soil cluster. The most important genes for the distinction of animal and plant host samples to the rest were genes linked to the COG category “*Replication, recombination and repair”* (for COG categories, see also Fig. 4). Genes most important for a shift towards the soil/sediment side of the plot were mainly linked to the COG categories “*Energy production and conversion”*, “*Lipid transport and metabolism”* and “*Replication, recombination and repair”*. The distinction between sediment and soil clusters was mainly due to genes linked to the COG categories “*Transcription”*, “*Xenobiotics biodegradation”* and “*Replication, recombination and repair”*. For the soil cluster the differentiating genes were assigned to the categories “*Xenobiotic biodegradation”* and “*Carbohydrate transport and metabolism”*, as well as “*Coenzyme transport and metabolism”* and “*Energy production”*. For 4 out of 19 genes of the 99% quantile explaining the clusters it was not possible to annotate any function. The list of genes of the 99% quantile which determine the differentiation between isolates from different ecosystems can be found in Table 1. When looking at the LDA of only genomes assigned to soil, we detected also here a clear separation of the microhabitats (data not shown). Genomes of rhizosphere isolates were clearly distinct from the rest, as well as isolates from natural soil sources (e.g., common garden soil, soil from polar regions and natural resorts). Genomes from contaminated soils build a cluster together with an agricultural soil. 30% of genomes from soil isolates could not be used for this more targeted LDA analysis, as metadata was not detailed enough.

**TABLE 1:** Genes of the 99% quantile determining the differentiation of genomes from different environments

Gene name	COG category	KO system	Annotation with Prokka
*thiC*	Coenzyme transport and metabolism	Metabolism of cofactors and vitamins	Phosphomethylpyrimidine synthase
group_745	Energy production and conversion	Carbohydrate metabolism	Putative 3-oxopropanoate dehydrogenase
*mdh*	Energy production and conversion	–	Alcohol dehydrogenase 2
*insK_1*	Replication, recombination, and repair	Unclassified: genetic information processing	Hypothetical protein
group_1838	Replication, recombination, and repair	Unclassified: genetic information processing	Hypothetical protein
group_5525	–	–	Hypothetical protein
*hsaB_3*	Function unknown	Xenobiotics biodegradation and metabolism	Flavin-dependent monooxygenase, reductase subunit *hsaB*
*xyR_1*	Transcription	Cellular processes	Putative hydrogen peroxide-inducible genes activator
*dprA*	Replication, recombination, and repair; intracellular trafficking, secretion, and vesicular transport	Unclassified: genetic information processing	Putative DNA processing protein *dprA*
group_870	Function unknown	–	Hydroxyacylglutathione hydrolase *gloC*
group_1919	Energy production and conversion	Unclassified: metabolism	S-(hydroxymethyl)mycothiol dehydrogenase
group_5103	Lipid transport and metabolism	–	Acyl-*CoA* dehydrogenase
group_6017	–	–	Hypothetical protein
*prpC*	Energy production and conversion	Carbohydrate metabolism	2-methylcitrate synthase
*fdhD*	Energy production and conversion	Unclassified: metabolism	Sulfur carrier protein *fdhD*
*dmpG*	Amino acid transport and metabolism	Xenobiotics biodegradation and metabolism, amino acid metabolism	4-hydroxy-2-oxovalerate aldolase
group_999	–	–	Hypothetical membrane protein
group_9090	–	–	Hypothetical protein
group_5673	Carbohydrate transport and metabolism; cell wall/membrane/envelope biogenesis	–	Hypothetical protein

### Genomic characterization of *R. pseudokoreensis* R79^T^ and *R. koreensis* R85

*R. pseudokoreensis* and *R. koreensis* cluster together with isolates from the other soil samples (Fig. S8). They shared a dDDH value of 57.9 and an ANI of 94.0%. Both strains showed large genome sizes compared to other *Rhodococcus* strains with 9.87 Mbp and a GC-content of 67.3% for *R. pseudokoreensis* R79^T^, and 10.91 Mbp with a GC-content of 67.2% for *R. koreensis* R85, respectively. Both strains had linear chromosomes and several mostly linear plasmids. For our isolates *R. pseudokoreensis* R79^T^ and *R. koreensis* R85, the annotation with Rapid Annotations using Subsystems Technology (RAST) revealed 9,521/10,868 coding DNA sequences (CDS) in total, with 60.4%/58.2% annotated features, and 21%/19% of all genes classified into 343/339 RAST subsystems in total, respectively. The number of RNAs was 62/64, respectively. Most CDS in the subsystems belonged to the RAST category “*Carbohydrates”* (22.8%/21.8%), followed by “*Amino Acids and Derivates”* (19.3%/21.4%), “*Fatty Acids, Lipids, and Isoprenoids”* (13.5%/13.6%), and “*Cofactors, Vitamins, Prosthetic Groups, Pigments”* (9.2%/9.0%). In general, both strains showed very similar gene patterns (Fig. S9). No resistance against known antibiotics could be found. Instead, resistance genes against heavy metals like cobalt, zinc, cadmium, copper, and mercury were present in both genomes. This was confirmed by the ResFinder and ToxFinder service, which could identify neither genes of acquired antibiotic resistance nor genes involved in mycotoxin synthesis for both strains.

**Fig 6:**
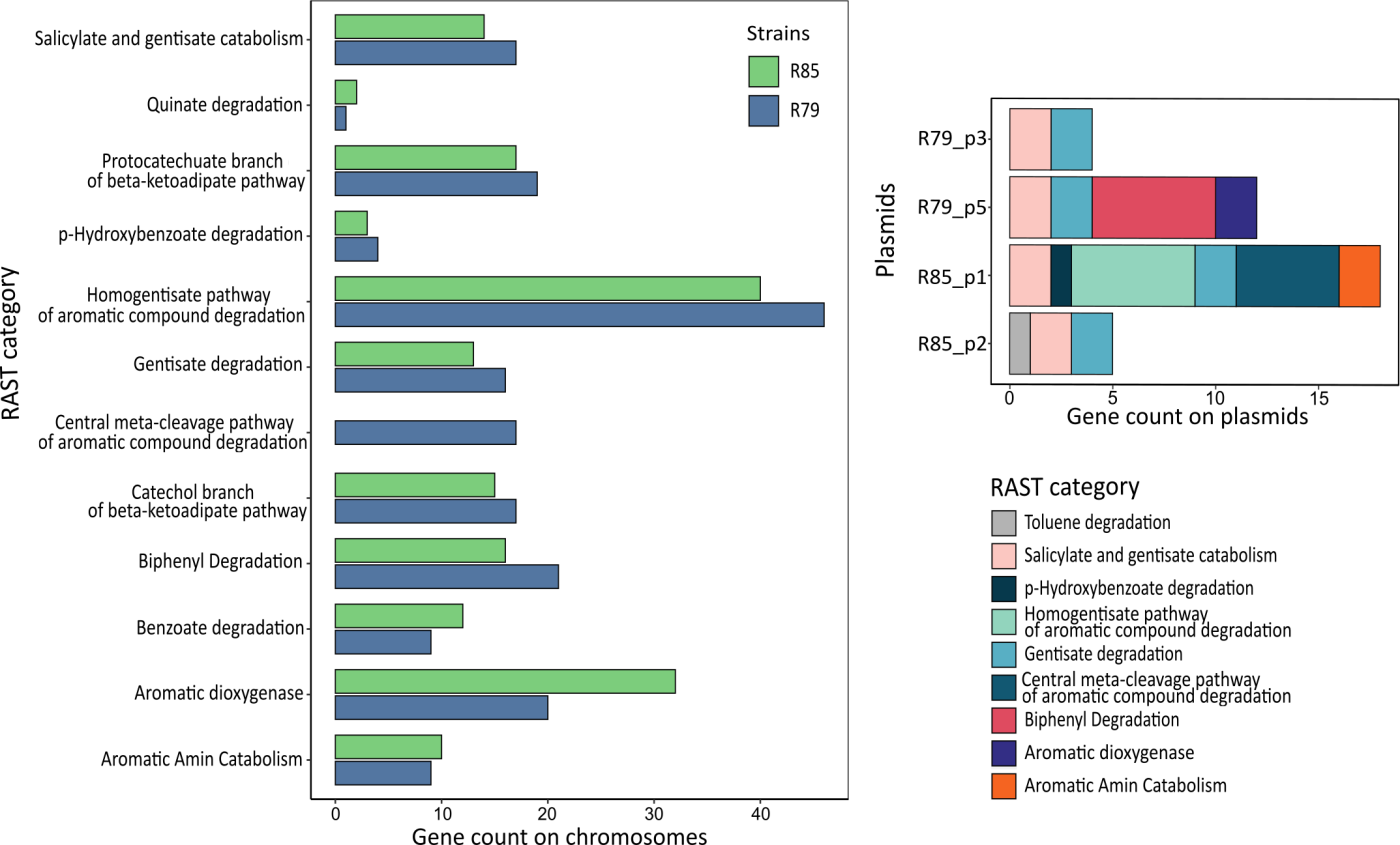
Abundance of genes per functional category encoding for the metabolism of aromatic compounds on the chromosomes and plasmids of isolated *R. pseudokoreensis* R79^T^ and closely related *R. koreensis* R85, annotated using the RAST server; only plasmids with respective degradation genes are displayed. The genes are not only located on plasmids, where they can act as fast mobile elements for horizontal gene transfer but also on chromosomes, ensuring higher stability for possible bioinoculation purposes.

Both strains were selected because of their potential ability to degrade aromatic compounds in soil. For R79^T^ and R85, 5.3% and 4.8% of genes, respectively, were annotated in RAST to subcategories encoding for the metabolism of aromatic compounds (Fig. 6). From the 12 RAST subcategories for metabolism of aromatic compounds displayed, most were present in similar amounts in both strains, except for the “Central meta-cleavage pathway of aromatic compound degradation” that was present on the chromosome only in R79^T^, while for R85 the respective genes were found in lower copy number only on plasmid R85_p1 (Fig. 5). For R79^T^, only two out of five plasmids harbored genes encoding for the metabolism of aromatic compounds, for R85 it was two out of four plasmids. Except for toluene degradation, which was only present on plasmid R85_p2, every subcategory encoding the metabolism of aromatic compounds on the plasmids occurred in higher gene numbers on the chromosome. As expected from *in vitro* tests, both strains had genes encoding for benzoate degradation like benzoate 1,2-dioxygenase subunit alpha and beta, with complete KEGG pathways for benzoate degradation, catechol ortho-cleavage, and catechol meta-cleavage. Both strains also harbored genes encoding for biphenyl degradation. R79^T^ possessed an almost complete pathway, only missing the last step of the degradation to benzoate or 4-chlorobenzoate.

**Fig 7:**
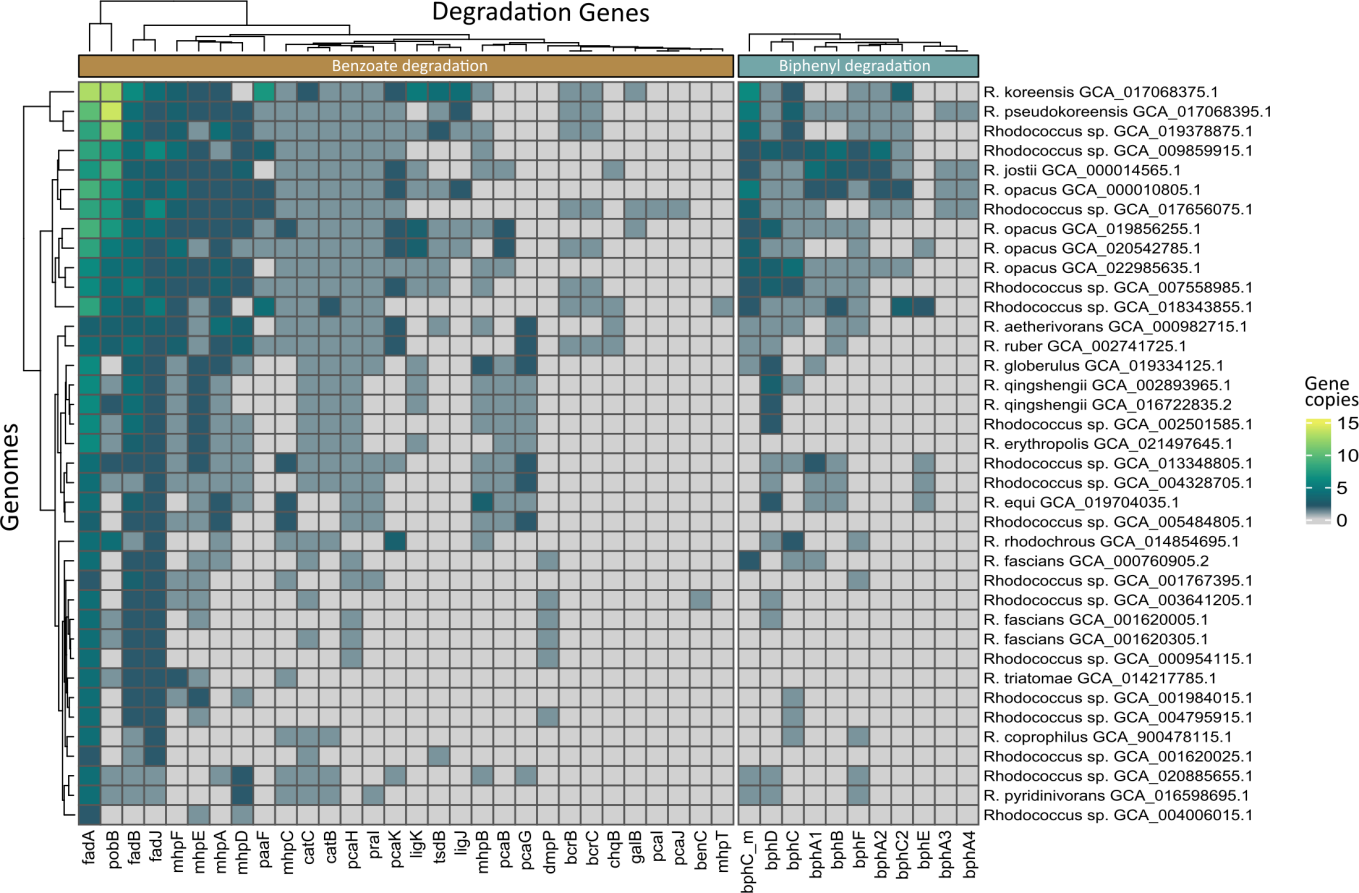
Heatmap of orthologous genes in 38 medoid *Rhodococcus* genomes coding for the degradation of benzoate and biphenyl. The number of paralogs was taken from the gene presence absence obtained with roary v3.13.0 and the heatmap was created with the R-package tidyHeatmap v1.10.0.

When comparing the ability of *R. pseudokoreensis* R79^T^ and *R. koreensis* R85 to degrade benzoate and biphenyl to other *Rhodococcus* members, it became obvious, that single genes important for especially benzoate degradation are spread throughout the whole genus (Fig. 7). Biphenyl degradation genes seemed to occur in higher abundance and more complete pathways in the genomes of strains closer related to *R. pseudokoreensis* and *R. koreensis*. However, single genes also were present in distantly related *Rhodococcus*.

## DISCUSSION

### *Rhodococcus* phylogenetic analysis and pan-genome

In this study, we performed comparative genomics on complete, high-quality genomes of the genus *Rhodococcus* to gain insights into the genetic diversity and evolutionary connections among its members. Our analysis revealed an open *Rhodococcus* pan-genome, with a growing number of accessory genes added with every new genome. The core was restricted to basic cell functioning, with the number of core genes (present in >95% of genomes) similar to the number of core genes for *Rhodococcus* found by Garrido-Sanz et al. 2020 (15). The pan-genome of the 109 genomes showed highly similar features to one of the 38 medoid genomes, indicating that the medoid genomes were a good representation of the genetic variance of the whole data set. We could not detect specific features in the *Rhodococcus* core genome, such as core virulence genes in *Campylobacter* (29). This underlines the large genomic and metabolic variability of the genus represented only in accessory genes. The big genomic differences between the respective species of *Rhodococcus* added to the ongoing debate about the reclassification of one or more phylogroups within *Rhodococcus* to new genera (21, 23).

In our data, we identified a number of potentially new *Rhodococcus* species by building new species-specific clades. As we only used complete genomes, most of the so far unclassified *Rhodococcus* strains built singleton species-specific clades. This is in accordance with Garrido-Sanz et al. 2020 (15) and confirms their assumption of many undescribed species in *Rhodococcus*. Furthermore, we saw several strains with the wrong taxonomic assignments. From our data, we concluded that even the frequently studied *R. jostii* RHA1 (11, 30), a degrader of polychlorinated biphenyl, could be an unknown species and not belong to *R. jostii*, as Jones et al. (31) stated. In our analysis, we found dDDH values of less than 42% compared to other *R. jostii* genomes. Although the GC content was very similar, the dDDH value was far below the common species cutoff of 70% genome similarity. This discrepancy could be due to different techniques employed for the analysis. Jones et al. (31) compared 16S rRNA sequences instead of whole genomes and determined the DNA:DNA relatedness in a laboratory approach. Their chemotaxonomic analysis hinted at *R. jostii*. However, Baek et al. (32) showed that chemotaxonomic properties have to be regarded with care and more confidence should be put into genomic analysis. Furthermore, we added on the work of Thompson et al. (33) and proposed the reclassification of additional *R. erythropolis* strains to *R. qingshengii* (Table S1).

Genome sizes of the strains differed by 6.33 Mbp from smallest to largest with a seemingly continuous distribution and no clustered size groups, suggesting no clear separation point of the genus based on genome size. It has been suggested to reclassify the relatively small genome-sized *R. equi* as a type species of a new genus called *Prescottella* (23, 34), together with closely related *Rhodococcus defluvii*, *Rhodococcus agglutinans*, *Rhodococcus soli,* and *Rhodococcus subtropica*. Other studies found *R. equi* to be deeply embedded within the phylogenetic tree of *Rhodococcus* and see it as a valid member of the genus (35, 36). We did see evidence for both, on the one hand, rhodococci with small genomes had down to only 34% of the size of the large *Rhodococcus* genomes, and shared a dDDH value of 19.8%, with a GC difference of 5.02%. This is in the range of differences to members of other genera, like *Streptomyces albireticli*, which shares 18.7% dDDH and a GC difference of 5.14% with *R. pseudokoreensis* (data not shown). However, *R. equi* strains do not have the smallest genomes in *Rhodococcus,* and removing them from the genus would leave a “gap” in the line of genome sizes. Consequently, the whole genus would need to be split up even more, like Sangal et al. (21) proposed, especially for the “*R. fascians* clade.” Creason et al. (37) found seven clades within *Rhodococcus* that shared an ANI value below 70%–75%, a threshold they consider significant to distinguish between different genera. In our study, we found only nine genomes that had ANI values lower than 77% (cutoff value of the fastANI program) compared to single other genomes. This is striking and could be explained with the use of different methods that over or underestimate ANI. However, Ciufo et al. (38) showed ANI values of 84%/82% between *Salmonella enterica* and *Escherichia coli*/*Enterobacter koseri*, demonstrating ANI values between different genera might be higher than Creason et al. proposed, also depending on the species defined in the genus. In our analysis, we saw nine phylogroups that shared ANI values of more than 80%. Seven of these groups were in line with the eight *Rhodococcus* phylogroups found by Sangal et al. 2019 (21). We did not identify the proposed *Rhodococcus maanshanensis*/*Rhodococcus tukisamuensis* group, as no members of these species were included in our analysis. Additionally, our data revealed a phylogroup with two unknown *Rhodococcus* strains from Antarctic soil (GCA_004795975, GCA_004795915), and a singleton, animal-associated strain with the smallest genome in the data set (GCA_004006015). Setting ANI and dDDH thresholds for taxonomic assignment to species to 95%/96% and 70%, respectively, is widely accepted. A threshold to distinguish between bacterial genera is still missing. However, this might be a very difficult task, as these values will vary a lot depending on the genus of interest, the quality of previous taxonomic assignments, and the method of analysis, as shown above.

In summary, we found high genetic variability within the genus *Rhodococcus*, together with enormous size differences in the genomes. We identified many undescribed species and several strains that need a reassessment of their taxonomic assignment. The huge diversity of *Rhodococcus* species suggests that splitting the genus into several genera might be justified. In general, a more unified concept for bacterial genera, especially for genome similarity, would be important to solve taxonomic problems.

### Functional analysis

To address if species affiliation alone determines the genetic similarity of the genomes, or if the isolation source is a triggering factor of the genome structure of the strains (H1), we analyzed the potential influence of habitat/isolation source on *Rhodococcu*s genomes using linear discriminant analysis. In accordance with Kumar et al. (39) who studied habitat-specific genes for the genus *Novosphingobium*, we found that the phylogenetic grouping of the strains differed significantly from clustering based on specific habitats. In addition, Kumar et al. also revealed strikingly large genomes and high gene content in environmental strains (39), which we also proved for *Rhodococcus*. This can be interpreted as important for these bacteria to adapt quickly to different habitats and changing nutrient conditions via differential gene expression, especially in environments such as soil (40). By applying LDA, we identified genes important for separating strains based on their isolation source. Genomes from plant, human, and animal host-associated strains formed distinct groups separated from each other and separated from strains isolated from soil. Animal host-associated strains isolated from different hosts (mammal, sponge, and insect) had comparatively small genomes and clustered very closely together, which is in line with findings of animal host-associated—mutualistic and pathogenic—bacteria sharing the same extracellular peptidase domain (41) and in general similar mechanism of genome reduction (42, 43). A strain isolated from contaminated groundwater clustered together with samples isolated from contaminated sites (sewage tank, bioreactor), suggesting a strong effect of environmental stress on structuring the genomes (44). While for the previously discussed clusters, common mechanisms apply, the genome of a strain isolated from marine water instead clustered together with the genomes of plant-associated strains, which might suggest similar genome reduction through different mechanisms. Pathogenic (in this case plant-associated) bacteria show a reduced genome (43), but not necessarily reduced especially in metabolically important genes like obligate endosymbionts, as it was thought before (45). However, many aquatic microbes also reduce their cells and genomes for effective use of energy and nutrients (46), according to the so-called streamlining theory (47). The genomes of our isolates from the apple rhizosphere clustered together with other strains isolated from the soil but not with the plant host-associated strains. Genes that differentiated genomes of plant host-associated strains from soil mainly belonged to the COG category “Replication, recombination, and repair,” while especially the samples isolated from soil and contaminated sites showed enrichment in genes important for primary and secondary metabolism. This was expected, as genomes of free-living strains are known to harbor more genes important for the regulation and biosynthesis of secondary metabolites, and fewer genes responsible for DNA replication, cell division, and nucleotide metabolism, compared to host-attached genomes (40).

For the discrimination of the strains based on habitat, there are two large pitfalls preventing in-depth analysis of genes important for differentiation. The first is the large number of unknown, unassigned genes in *Rhodococcus*. The second is the huge inconsistency in the quality of metadata provided with the genome sequences in the databases (48). To facilitate targeted analysis of microbial pan-genomes and the influence of different factors on the genetic repertoire of the taxa, we need to stress the importance of providing exhaustive and accurate metadata upon submission of genomic sequences. For further analysis of the evolutionary history of the genomes, especially of the soil isolates, follow-up studies might examine the megaplasmids of the *Rhodococcus* pan-genome, to identify key features and possible integration events, in addition to those described for the larger chromosomes.

### Genomic characterization of *R. pseudokoreensis* R79^T^ and *R. koreensis* R85

Genomes of both strains *R. pseudokoreensis* R79^T^ and *R. koreensis* R85 isolated from apple rhizosphere were similar with respect to genome size, GC-content, ANI, dDDH, and annotated genetic features, supporting their close taxonomic relationship (26). The isolates harbored several linear plasmids and one circular plasmid each (27). This mix of replicon structures was also found for other rhodococci (49). Other than for circular plasmids, mechanisms of linear plasmid transfer in actinomycetes and other bacteria have not been intensively investigated (50). However, it is known that horizontal gene transfer (HGT) via linear replicons plays an important role in spreading metabolically important genes within the genus *Rhodococcus* (49), and from *Rhodococcus* spp. to other Gram-positive genera (51). Moreover, genetic bioaugmentation via plasmid transfer was shown to help in degrading harmful substances from the environment (52).

Both isolates harbored genes for the complete pathways for benzoate degradation, catechol ortho- and meta-cleavage, and genes for biphenyl degradation. These and other phenolic compound degradation genes are located on the chromosomes, apart from the central meta-cleavage pathway for R85. Some of these genes like biphenyl degradation genes and other aromatic dioxygenases for R79^T^ are additionally located on the linear plasmids. Sun et al. 2023 (53) found two different mechanisms of phenol degradation within the same bacterial species, a single-pathway pattern with degradation genes located on the chromosomes and a dual-pathway pattern with additional genes on the plasmids. This dual-pathway pattern could be expressed in high phenol concentrations, was superior in degradation performance, and could be transferred to other strains via HGT (53). This might be a mechanism also employed in our *Rhodococcus* isolates. Harboring the potential for both, a functional ortho- and meta-cleavage pathway again promotes a higher metabolic flexibility of the strains. This is also known for *Pseudomonas putida*, which can induce simultaneous degradation via the ortho- and meta-cleavage pathway if the benzoate concentration is high (54). The transcriptional regulation of genes for biphenyl degradation is induced by the presence of the substance itself (55). Taguchi et al. 2007 (56) found the sequence and the arrangement of biphenyl degradation genes to be different among members of *Rhodococcus* and an indication for recombination around responsible gene clusters. From this, Fujihara et al. (57) concluded that biphenyl degradation gene clusters evolved independently and spread through HGT within the genus. Genes important for the biosynthesis of phytoalexins, especially biphenyls, were found to be upregulated in plants grown in replant-affected soil (24, 58) but not with other abiotic stresses (59). Benzoic acid, phlorizin, and vanillic acid were the major types of phenolic compounds found in higher concentrations in ARD soil (60). Duan et al. (61) showed in their study, that *Bacillus licheniformis* XNRB-3 could enhance plant growth and the activity of disease resistance-related enzymes while lowering the phenolic content (mainly phlorizin) in the rhizosphere. Also, Jiang et al. (62) found a positive effect on apple growth inoculating with a phenolic acids degrading strain. Compared to other members of the genus, our isolates showed a higher copy number and in general more genes related to the degradation of benzoate and biphenyl, also in accordance with the trend of large genomes being disproportionately enriched in secondary metabolite genes (40). Although members of *Rhodococcus* are known for their exceptional degradation abilities, isolates R79 and R85 harbored more genes related to the degradation of benzoate and biphenyl than any other strain of this study. In a few, large genomes (including R79^T^ and R85), we detected *bcrB* and *bcrC* genes (Fig. 7), which could indicate the presence of benzoyl-coenzyme A reductase involved in anaerobic degradation of aromatic compounds (63, 64). Most rhodococci are known to be aerobic, however, some strains were proven to be facultative anaerobic (65, 66). This implies that the strains harboring the *bcrBC* genes could indeed be facultative anaerobic and switch their activity according to the environmental conditions.

The presented features might enable *R. pseudokoreensis* R79^T^ and *R. koreensis* R85 to mitigate ARD by alleviating the stress possibly induced by phenolic compounds in the soil. Additionally, the position of the genes on the linear chromosome and the linear plasmid may also include the possible spreading of degradation genes via HGT to other soil bacteria, which might be a further advantage for the application. However, this along with their applicability against ARD needs to be further tested in greenhouse- and field experiments.

## MATERIALS AND METHODS

### Molecular characterization of isolates and genome sequencing

Two actinobacterial strains, isolated from the rhizosphere of young apple plantlets grown on natural soil, were selected because of their ability to degrade benzoate (data not shown). Details on the isolation of the strains, extraction of genomic DNA, genome sequencing, and genome assembly, as well as information about chromosome and plasmid structures, were described in the genome announcement of Benning et al. 2021 (27). A precise taxonomic affiliation was inferred from the whole genome sequences using the Type (Strain) Genome Server (TYGS) (67). Isolate R85 was identified as *Rhodococcus koreensis* with dDDH values of 87.5% and 87.2% compared to *R. koreensis* NBRC 100607 and DSM 44498, respectively. The genome sequence of R85 is the only complete sequence of *R. koreensis* that is currently available (13.06.2023) on NCBI and therefore used as a reference genome (RefSeq) for this species. Isolate R79^T^ was identified as a new *Rhodococcus* species (<58.0% dDDH for all comparisons) and described as a type strain of *R. pseudokoreensis* in Kämpfer et al. (26). Annotation was done using Rapid Annotation using Subsystem Technology (RAST, v.2.0) (68), with default parameters. Additionally, the ResFinder and ToxFinder service of the Center for Genomic Epidemiology (https://www.genomicepidemiology.org/services/; visited 7 March 2023) was used to screen for genes of acquired antibiotic resistance and genes involved in mycotoxin synthesis. Analysis and figures were done in R v.4.2.2 (69).

### Selection of *Rhodococcus* model genomes

For comparison with *Rhodococcus* strains R85 and R79, all complete, high-quality genomes of *Rhodococcus* were downloaded from the Reference Sequence (RefSeq) database at NCBI (ftp://ftp.ncbi.nih.gov/genomes/, accessed on 21 June 2022). The quality of the genomes was controlled using CheckM v.1.2.0 (70). Out of 110 downloaded genomes (including genomes of R79 and R85), 109 were selected with completeness >97.5%, contamination <4.62%, and maximum of seven contigs. Based on metadata, the genomes included in this study originated from diverse habitats, spanning abiotic habitats like water and soil—including highly contaminated sites—as well as human, animal, and plant hosts. Detailed information about accession numbers, taxonomy, and strain names is given in Table S2, and information about isolation sources is provided in Table S3.

### Pan-genome construction and phylogenetic analysis

For pan-genome construction, all downloaded genome sequences were annotated using Prokka v.1.13 (71). From the resulting GFF3 files, genes were grouped into orthologous gene clusters with roary v.3.13.0 (72) and identified as core, shell, and accessory genes of the pan-genome. The minimum percentage identity for BLASTp searches was 70% and orthologous genes were clustered by the Markov cluster algorithm with an inflation value of 1.5. Results were visualized with a custom roary python script and the R-package Pagoo v.0.3.18. A phylogenetic tree was built, inferring approximately maximum-likelihood phylogeny from the single-copy core genome alignment from roary using FastTree2 (73). Visualization and addition of metadata were done in iTOL v.6.7.3 (74). dDDH values for the genomes were calculated using the Type (Strain) Genome Server TYGS (67). All dDDH values given in this study are based on the formula d_4_, the preferred method of TYGS, as it is independent of genome length (67).

### Functional analysis for 38 medoid genomes

To level the number of strains coming from different *Rhodococcus* species in the data set, symmetric pairwise ANI dissimilarities were calculated from the whole genome sequences with fastANI v.1.32 (75). Medoid genomes (most centrally located genomes of the cluster) were selected for a 98% similarity threshold using the hclust function as implemented in the R package bactaxR v.0.2.2 (76) based on ANI dissimilarities. Only those strains with less than 98% ANI similarity were kept for analysis as medoid genomes. Comparison between the analysis of all 109 genomes and the analysis of the resulting 38 medoid genomes showed that no diversity was lost and that the 38 medoid genomes were a good representation of the overall variability.

For search against the COG database, genomes were annotated using Prodigal v2.6.3 (77). Functional annotation against COG was done using eggnogg-mapper v.2.1.9 (78) and the EggNOG database v.5.0 orthology assignments (79).

LDA was performed in R to predict the impact of single genes on different model response variables like isolation source/habitat or phylogeny. We used R-package MASS v.7.3-58.1 with function “model <- lda(data[ ,4] ~ ., data = total[ ,8:ncol(data)]).” After filtering the gene presence–absence data from roary to exclude low variance variables, 920 out of 63,064 genes had a standard deviation >0.5 and were included in the analysis.

To compare the genetic ability for degradation of aromatic compounds, presence–absence matrices, and heatmaps were constructed in R with R-packages ggplot2 v.3.4.2 (80) and tidyHeatmap v.1.10.0 (81), respectively, using the pan-genome gene presence–absence data from roary. Genes were selected according to the 21 KEGG database pathways for *xenobiotics biodegradation and metabolisms* (https://www.genome.jp/kegg/pathway.html), with focus on biphenyl and benzoate degradation.

## Data Availability

The full genome sequences of *R. pseudokoreensis* R79^T^ and *R. koreensis* R85 have been deposited in GenBank under BioProject number PRJNA700828. The accession numbers are CP070614 to CP070619 for R79 and CP070609 to CP070613 for R85. Raw reads have been deposited in the Sequence Read Archive (SRA) with accession numbers SRX10094454 for R79 and SRX10094455 for R85. *R. pseudokoreensis* R79 is available from culture collections as DSM 113102^T^, LMG 32444^T^ and CCM 9183^T^; and *R. koreensis* R85 is available as DSM 116493.

## SUPPLEMENTAL MATERIAL

Fig. S1 to S9 and Tables S1 to S3 spectrum.03783-23-s0001.pdf Supplementary material for article combined in one PDF.

ASM does not own the copyrights to Supplemental Material that may be linked to, or accessed through, an article. The authors have granted ASM a non-exclusive, world-wide license to publish the Supplemental Material files. Please contact the corresponding author directly for reuse.
