# Supplementary material for: (Pan)genomic analysis of two Rhodococcus isolates and their role in phenolic compound degradation
Source: Microbiol Spectr. 2024 Feb 20;12(4):e03783-23. doi: 10.1128/spectrum.03783-23 (PMC10986565; doi:10.1128/spectrum.03783-23)
Supplement: Fig. S1 to S9 and Tables S1 to S3 — Supplementary material for article combined in one PDF. [file spectrum.03783-23-s0001.pdf]

Supplementary file for (Pan)genomic analysis of two *Rhodococcus* isolates and their role in phenolic compound degradation

Sarah Benning,[a]# Karin Pritsch,[b] Viviane Radl,[a]* Roberto Siani,[a] Zhongjie Wang,[a] Michael Schloter[c,a]

[a]Research Unit for Comparative Microbiome Analysis, Helmholtz Munich, German Research Center for Environmental Health, Neuherberg, Germany

[b]Research Unit for Environmental Simulations, Helmholtz Munich, German Research Center for Environmental Health, Neuherberg, Germany

[c]Chair for Environmental Microbiology, TUM School of Life Sciences Technical University Munich, Germany

Running Head: Pan-genome study of *Rhodococcus*

Adress correspondence to Sarah Benning, sarah.benning@helmholtz-munich.de

*Present address: Viviane Radl, Department of Safety and Area Management, Helmholtz Munich, German Research Center for Environmental Health, Neuherberg, Germany

Supplementary Figures

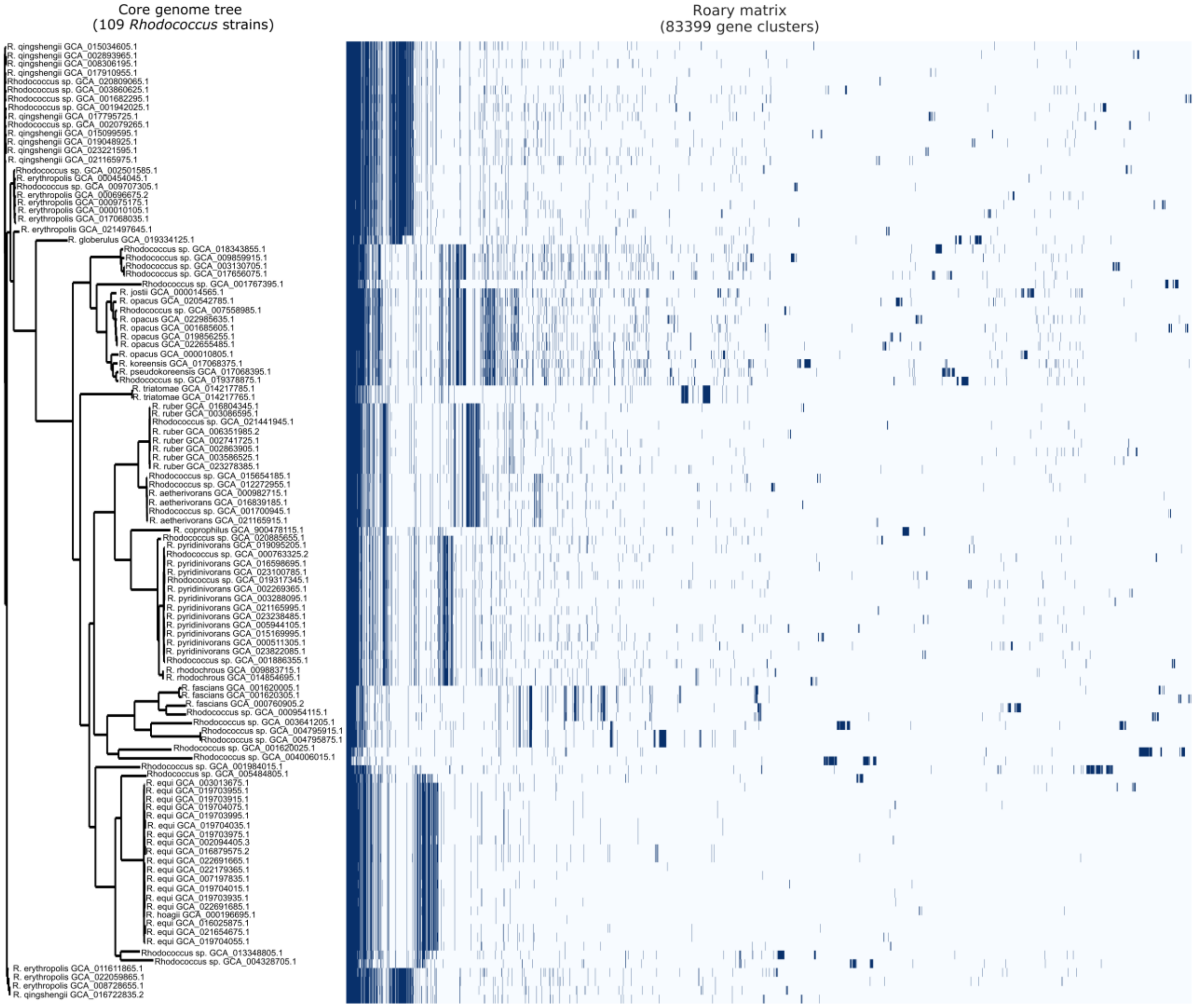

Supplementary Figure S 1. Gene Presence / Absence binmap showing cluster of orthologues genes per genome. Shown are 109 complete *Rhodococcus* genomes retrieved from NCBI database. The binmap was constructed on the base of the gene presence absence matrix obtained with roary v3.13.0 with parameter blastp identity = 70 % and MCL inflation value = 1.5. The phylogenetic tree was build with FastTree, inferring approximately-maximum-likelihood phylogeny using the core gene alignment constructed by roary.

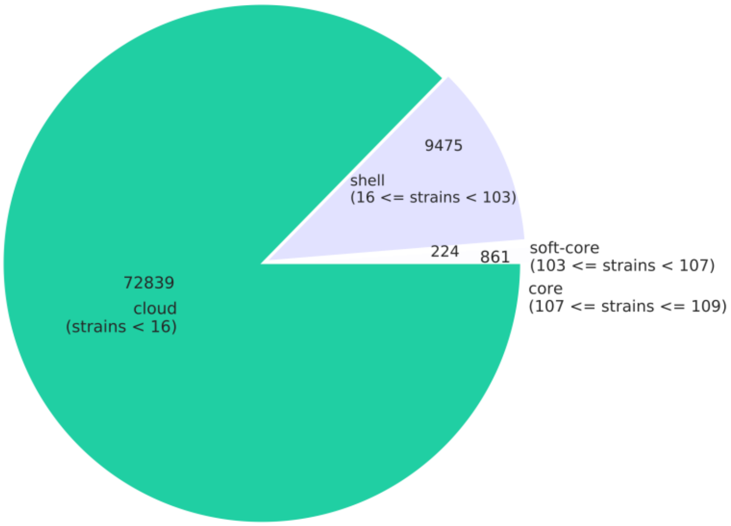

Supplementary Figure S 2. Pangenome-pie. Number of core, shell and cloud gene cluster of 109 selected Rhodococcus genomes constructed using the gene presence absence matrix obtained with roary v. 3.13.0. Parameter for roary were minimum percentage identity for blastp = 70 and MCL inflation value = 1.5. Core / soft core genes are present in 99 % / 95 % of all strains, shell genes are present in at least 15 % and cloud or accessory genes are the remaining genes, present only in one or very few genomes.

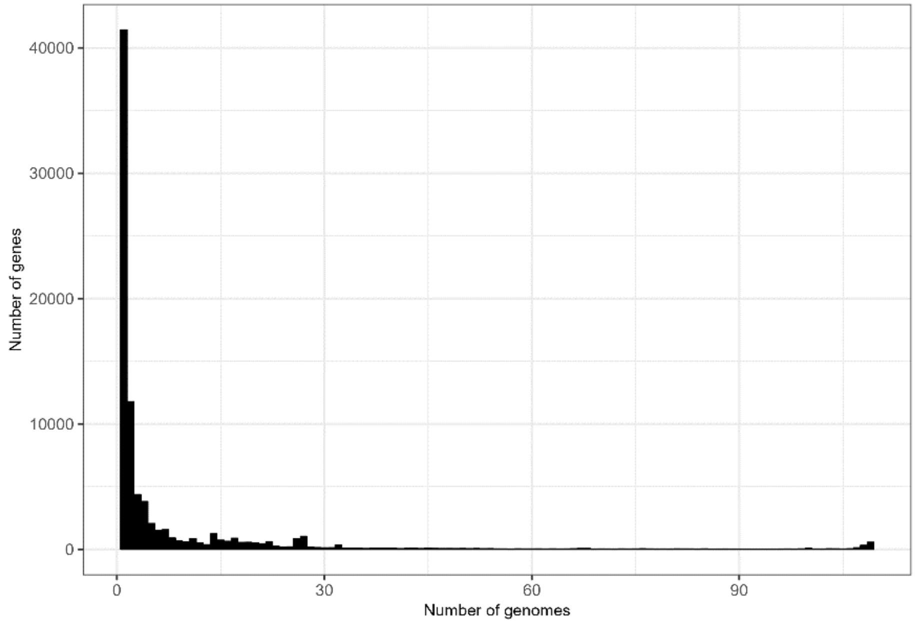

Supplementary Figure S 3. Frequency barplot of gene cluster vs. number of genomes. It is indicated, how many genes are present in one genome, two genomes... to all 109 genomes, which represents the core. The graph was produced using the R package pagoo v. 0.3.13 and the gene presence absence matrix obtained with roary v. 3.13.0. Parameter for roary were minimum percentage identity for blastp = 70 and MCL inflation value = 1.5. The amount of genes unique to one or only several strains is very high, the core is reduced to basic cell functioning

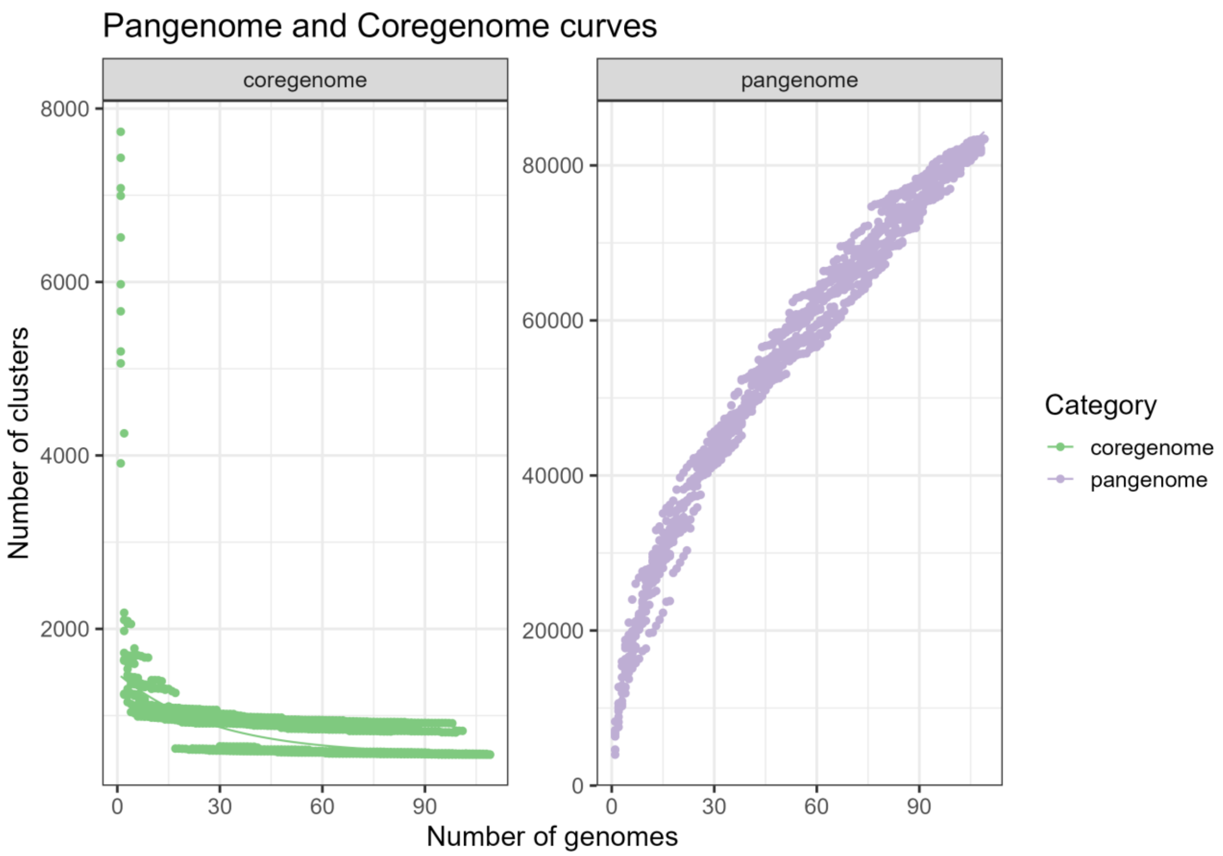

Supplementary Figure S 4. Pan-genome curves for 109 *Rhodococcus* genomes. The pan-genome is open. The graph was produced using the R package pagoo v. 0.3.13 and the gene presence absence matrix obtained with roary v. 3.13.0.

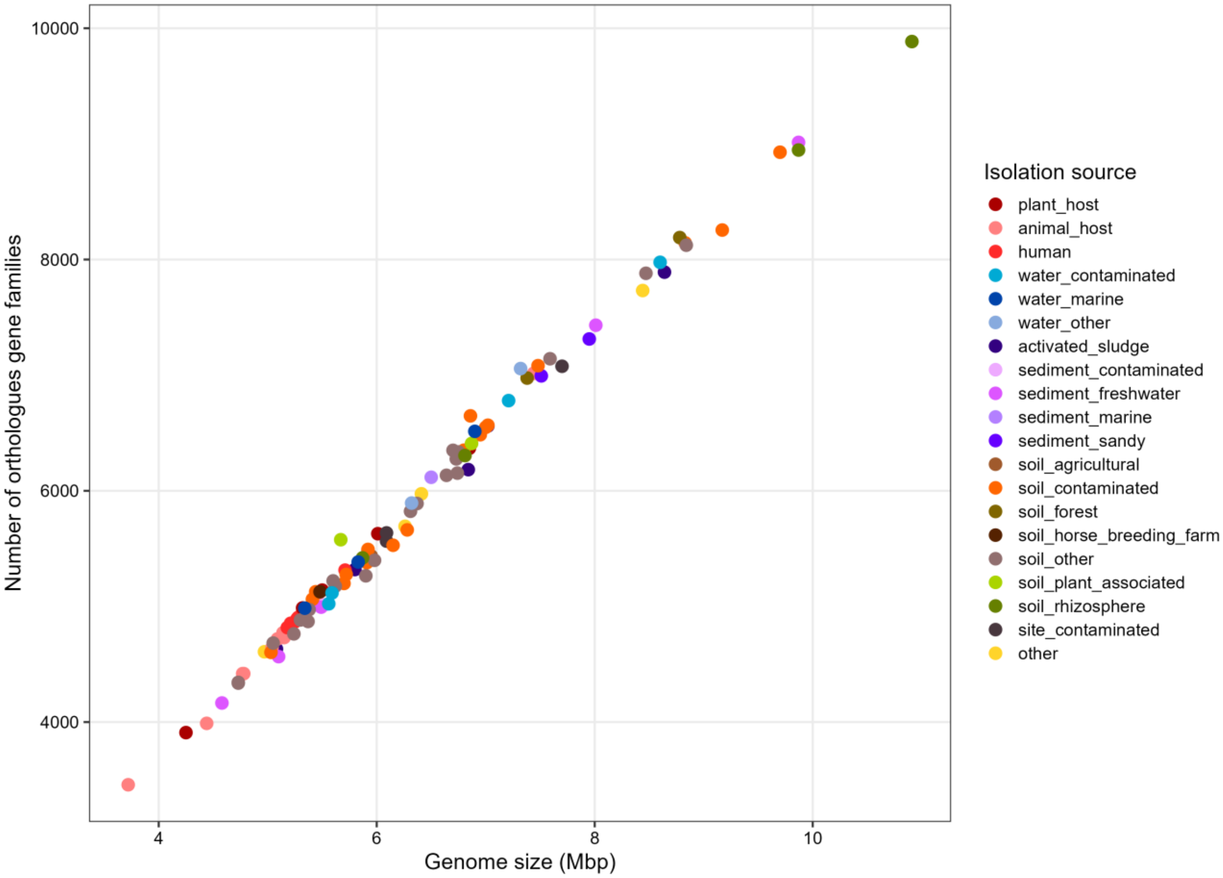

Supplementary Figure S 5. Plot of genome size vs. number of orthologues gene families per strain, coloured by isolation source. Number of orth. gene families was calculated from the gene presence absence matrix retrieved from roary roary v. 3.13.0. There is grouping according to habitat, host attached strains tend to have smaller genomes. However, soil and sediment strains are spread throughout all genome sizes.

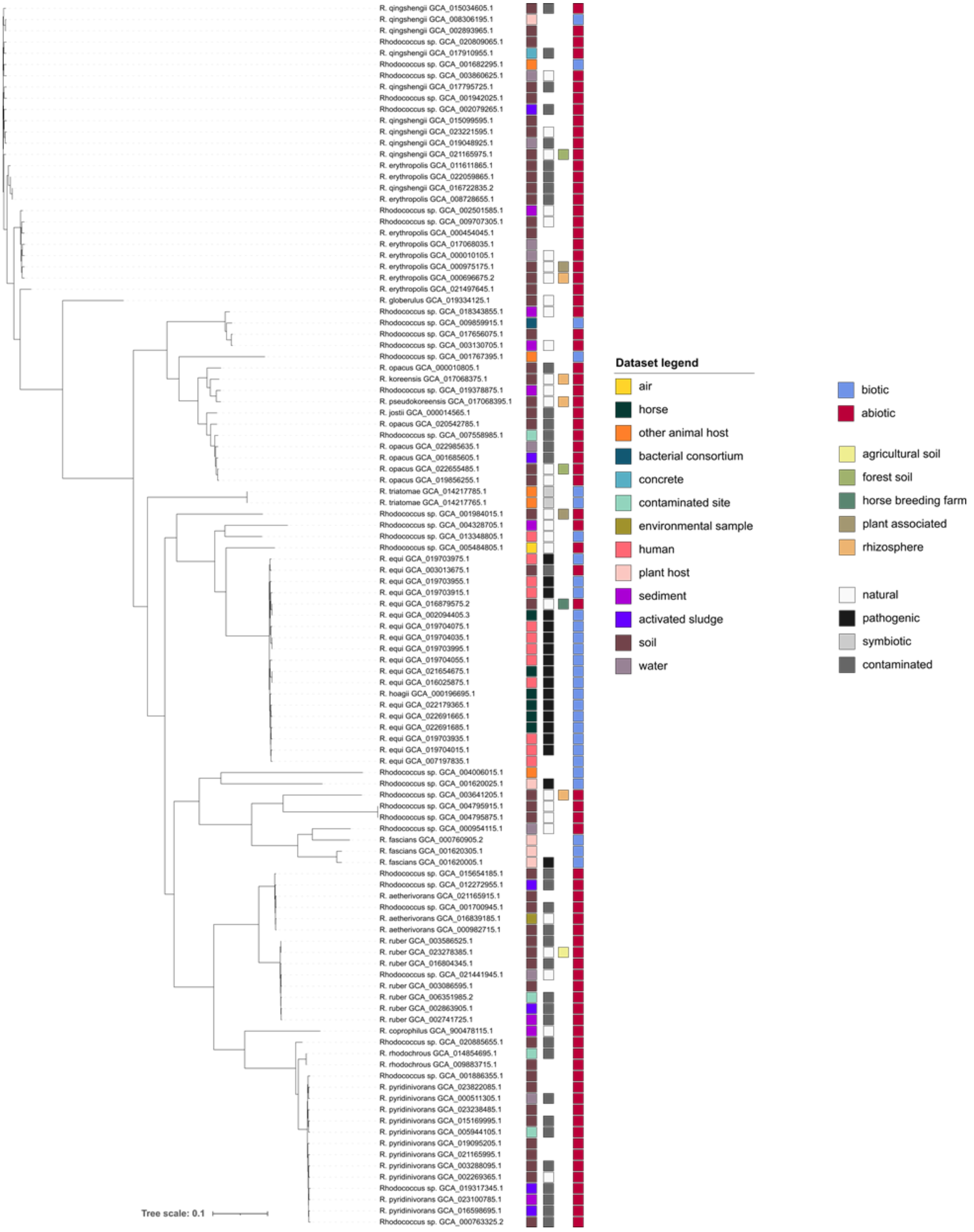
Tree scale: 0.1
Dataset legend
air
horse
other animal host
bacterial consortium
concrete
contaminated site
environmental sample
human
plant host
sediment
activated sludge
soil
water
biotic
abiotic
agricultural soil
forest soil
horse breeding farm
plant associated
rhizosphere
natural
pathogenic
symbiotic
contaminated

54

Supplementary Figure S 6. Phylogenetic tree of all 109 selected genomes calculated from the core genome alignment derived from roary v. 3.13.0 using FastTree (1). Visualisation and metadata adding was done in iTOL v. 6.7.3 (2). The metadata added gives information about the isolation source of the strains and mode of life, biotic = host attached, abiotic = free living strain; missing value means unknown status

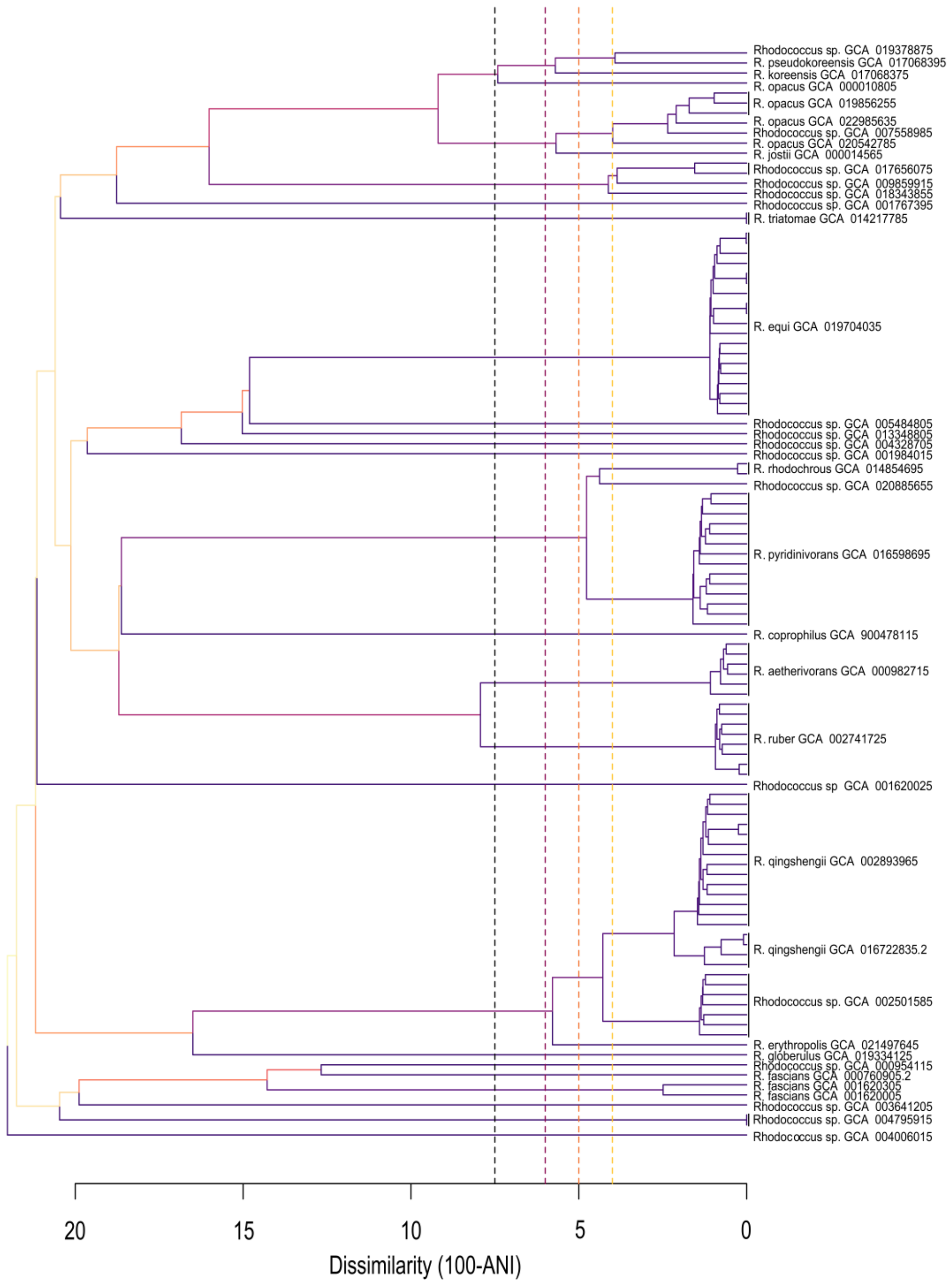

Supplementary Figure S 7. Dendrogram of 109 *Rhodococcus* genomes from the NCBI database, constructed using hclust function as implemented in the R package bactaxR v. 0.2.2 based on symmetric pairwise average nucleotide identity (ANI) dissimilarities calculated with fastANI 1.32. Displayed 38 strains are respective medoid genomes at 98% similarity threshold,

dashed lines represent dissimilarity values of 4, 5, 6 and 7.5, which corresponds to ANI values of 96, 95, 94 and 92.5 percent similarity respectively, as per default in bactaxR. Colour of branches corresponds to respective lenght in the tree.

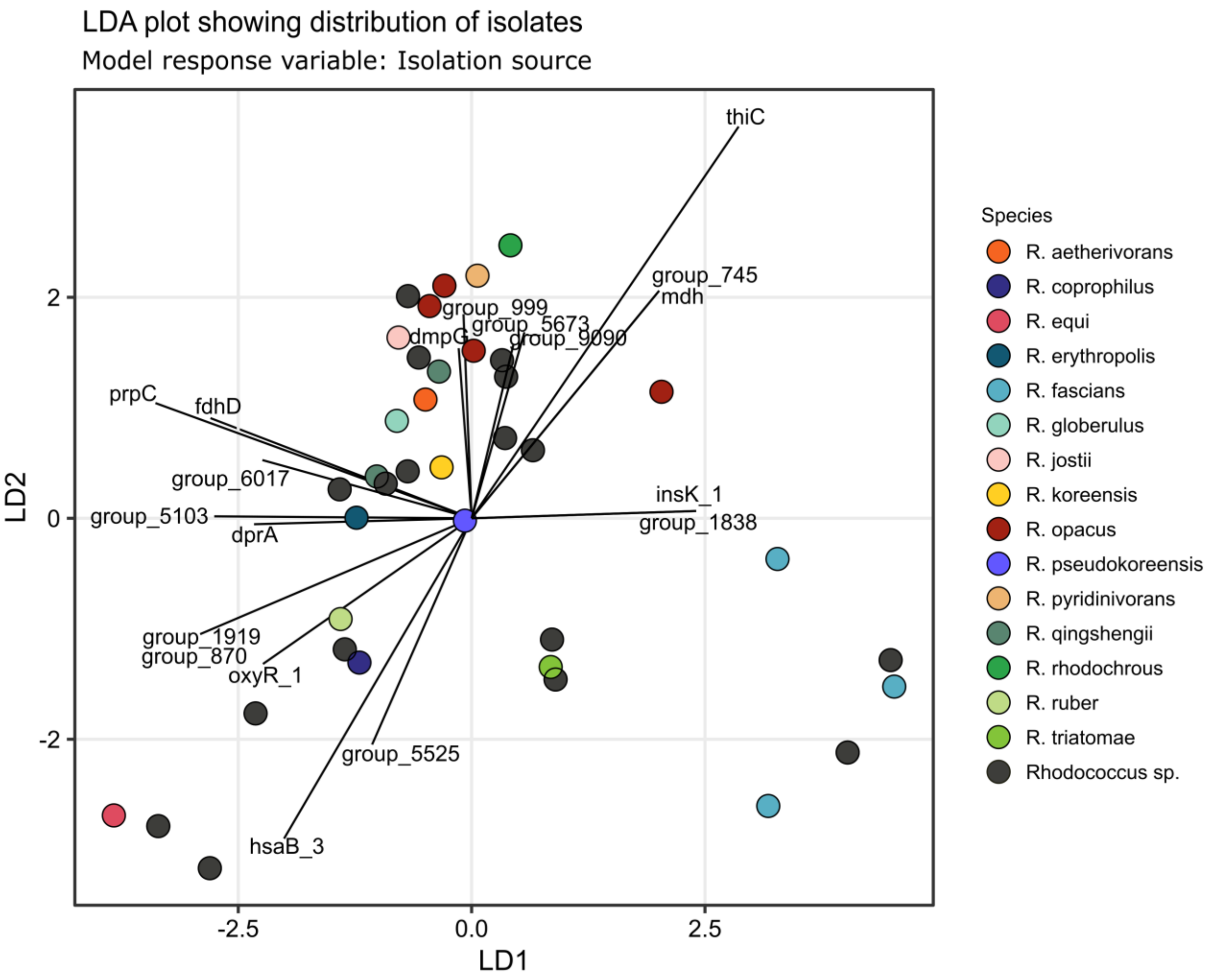

Supplementary Figure S 8. Linear Discriminant Analysis (LDA) plot of genes of 38 medoid genomes. Model response variable: Isolation source. Only genes of the 99% quantile shown

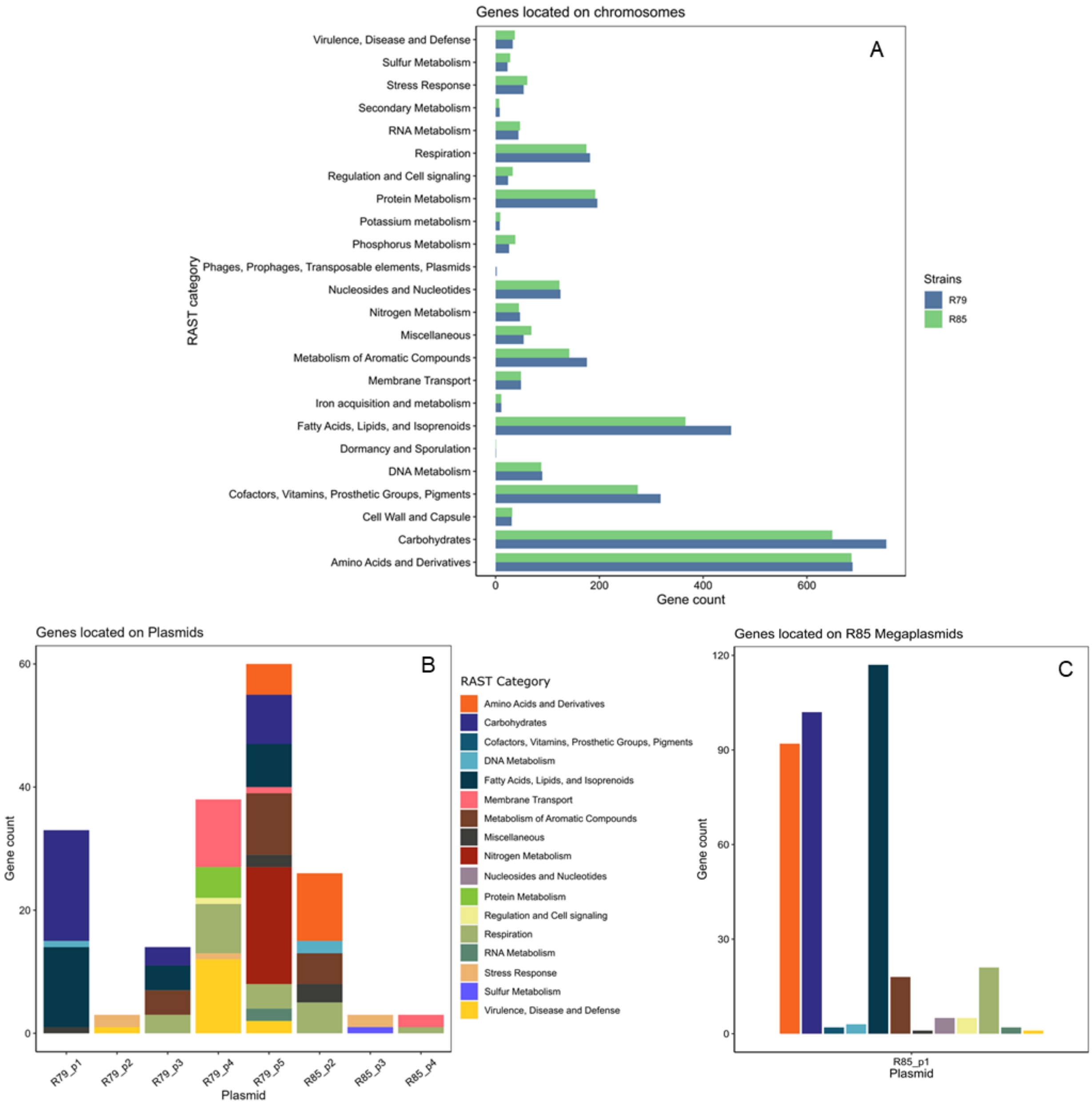

Supplementary Figure S 9. Abundance of genes per functional category located on (A) the chromosomes (genomes without plasmids) and on the several plasmids of *Rhodococcus pseudokoreensis* R79 *and R. koreensis* R85 with (B) all plasmids of *R. pseudokoreensis* R79 and small plasmids of *R. koreensis* R85, (C) megaplasmid of R85, annotated using the RAST (Rapid Annotations using Subsystems Technology) server v. 2.0

Supplementary Tables

Supplementary Table S1. All genomes with uncertain taxonomic affiliation and proposed new designation

GENBANK ASSEMBLY ID	SPECIES	STRAIN	NEW DESIGNATION
GCA_008728655.1	*R. erythropolis*	X5	*R. qingshengii*
GCA_011611865.1	*R. erythropolis*	KB1	*R. qingshengii*
GCA_021497645.1	*R. erythropolis*	D310-1	potentially new species
GCA_022059865.1	*R. erythropolis*	CERE8	*R. qingshengii*
GCA_000760905.2	*R. fascians*	A21d2	potentially new species
GCA_000014565.1	*R. jostii*	RHA1	potentially new species
GCA_000010805.1	*R. opacus*	B4	potentially new species
GCA_000763325.2	*Rhodococcus* sp.	p52	*R. pyridinivorans*
GCA_000954115.1	*Rhodococcus* sp.	B7740	potentially new species
GCA_001620025.1	*Rhodococcus* sp.	PBTS 1	*R. kroppenstedtii*
GCA_001682295.1	*Rhodococcus* sp.	008	*R. qingshengii*
GCA_001700945.1	*Rhodococcus* sp.	WB1	*R. aetherivorans*
GCA_001767395.1	*Rhodococcus* sp.	WMMA185	potentially new species
GCA_001886355.1	*Rhodococcus* sp.	2G	*R. pyridinivorans*
GCA_001942025.1	*Rhodococcus* sp.	YL-1	*R. qingshengii*
GCA_001984015.1	*Rhodococcus* sp.	MTM3W5.2	potentially new species
GCA_002079265.1	*Rhodococcus* sp.	BH4	*R. qingshengii*
GCA_002501585.1	*Rhodococcus* sp.	H-CA8f	*R. erythropolis*
GCA_003130705.1	*Rhodococcus* sp.	S2-17	*R. oxybenzonivorans*
GCA_003641205.1	*Rhodococcus* sp.	P1Y	potentially new species
GCA_003860625.1	*Rhodococcus* sp.	NJ-530	*R. qingshengii*
GCA_004006015.1	*Rhodococcus* sp.	X156	potentially new species
GCA_004328705.1	*Rhodococcus* sp.	ABRD24	potentially new species
GCA_004795875.1	*Rhodococcus* sp.	PAMC28705	potentially new species x
GCA_004795915.1	*Rhodococcus* sp.	PAMC28707	potentially new species x
GCA_005484805.1	*Rhodococcus* sp.	SGAir0479	potentially new species

GCA_007558985.1	*Rhodococcus* sp.	WB9	*R. opacus*
GCA_009707305.1	*Rhodococcus* sp.	AQ5-07	*R. erythropolis*
GCA_009859915.1	*Rhodococcus* sp.	WAY2	*R. oxybenzonivorans*
GCA_012272955.1	*Rhodococcus* sp.	DMU1	*R. aetherivorans*
GCA_013348805.1	*Rhodococcus* sp.	W8901	potentially new species
GCA_015654185.1	*Rhodococcus* sp.	M8	*R. aetherivorans*
GCA_017656075.1	*R. oxybenzonivorans*	ZPP	type strain, species described after data dowload
GCA_018343855.1	*Rhodococcus* sp.	USK13	*R. oxybenzonivorans*
GCA_019317345.1	*Rhodococcus* sp.	LW-XY12	*R. pyridinivorans*
GCA_019378875.1	*Rhodococcus* sp.	USK10	*R. pseudokoreensis*
GCA_020809065.1	*Rhodococcus* sp.	C1	*R. qingshengii*
GCA_020885655.1	*Rhodococcus* sp.	RDE2	potentially new species
GCA_021441945.1	*Rhodococcus* sp.	DMF-1	*R. ruber*

Supplementary Table S2. GenBank assembly numbers and species names for all genomes used in this study. It is indicated if they were part of the 38 medoid genomes with more than 98% genome dissimilarity

GENBANK ASSEMBLY ID	SPECIES	STRAIN	38_MEDOID
GCA_000010105.1	*Rhodococcus erythropolis*	PR4	0
GCA_000010805.1	*Rhodococcus opacus*	B4	1
GCA_000014565.1	*Rhodococcus jostii*	RHA1	1
GCA_000196695.1	*Rhodococcus hoagii*	103S	0
GCA_000454045.1	*Rhodococcus erythropolis*	CCM2595	0
GCA_000511305.1	*Rhodococcus pyridinivorans*	SB3094	0
GCA_000696675.2	*Rhodococcus erythropolis*	R138	0
GCA_000760905.2	*Rhodococcus fascians*	A21d2	1
GCA_000763325.2	*Rhodococcus sp. p52*	p52	0
GCA_000954115.1	*Rhodococcus sp. B7740*	B7740	1
GCA_000975175.1	*Rhodococcus erythropolis*	BG43	0
GCA_000982715.1	*Rhodococcus aetherivorans*	IcdP1	1
GCA_001620005.1	*Rhodococcus fascians*	PBTS 2	1
GCA_001620025.1	*Rhodococcus sp. PBTS 1*	PBTS 1	1
GCA_001620305.1	*Rhodococcus fascians*	D188	1
GCA_001682295.1	*Rhodococcus sp. 008*	008	0
GCA_001685605.1	*Rhodococcus opacus*	1CP	0
GCA_001700945.1	*Rhodococcus sp. WB1*	WB1	0
GCA_001767395.1	*Rhodococcus sp. WMMA185*	WMMA185	1
GCA_001886355.1	*Rhodococcus sp. 2G*	2G	0
GCA_001942025.1	*Rhodococcus sp. YL-1*	YL-1	0
GCA_001984015.1	*Rhodococcus sp. MTM3W5.2*	MTM3W5.2	1
GCA_002079265.1	*Rhodococcus sp. BH4*	BH4	0
GCA_002094405.3	*Rhodococcus equi*	PAM2287	0
GCA_002269365.1	*Rhodococcus pyridinivorans*	GF3	0
GCA_002501585.1	*Rhodococcus sp. H-CA8f*	H-CA8f	1
GCA_002741725.1	*Rhodococcus ruber*	P14	1

GCA_002863905.1	*Rhodococcus ruber*	YYL	0
GCA_002893965.1	*Rhodococcus qingshengii*	djl-6-2	1
GCA_003013675.1	*Rhodococcus equi*	DSSKP-R-001	0
GCA_003086595.1	*Rhodococcus ruber*	SD3	0
GCA_003130705.1	*Rhodococcus sp. S2-17*	S2-17	0
GCA_003288095.1	*Rhodococcus pyridinivorans*	TG9	0
GCA_003586525.1	*Rhodococcus ruber*	YC-YT1	0
GCA_003641205.1	*Rhodococcus sp. P1Y*	P1Y	1
GCA_003860625.1	*Rhodococcus sp. NJ-530*	NJ-530	0
GCA_004006015.1	*Rhodococcus sp. X156*	X156	1
GCA_004328705.1	*Rhodococcus sp. ABRD24*	ABRD24	1
GCA_004795875.1	*Rhodococcus sp. PAMC28705*	PAMC28705	0
GCA_004795915.1	*Rhodococcus sp. PAMC28707*	PAMC28707	1
GCA_005484805.1	*Rhodococcus sp. SGAir0479*	SGAir0479	1
GCA_005944105.1	*Rhodococcus pyridinivorans*	YF3	0
GCA_006351985.2	*Rhodococcus ruber*	R1	0
GCA_007197835.1	*Rhodococcus equi*	WY	0
GCA_007558985.1	*Rhodococcus sp. WB9*	WB9	1
GCA_008306195.1	*Rhodococcus qingshengii*	RL1	0
GCA_008728655.1	*Rhodococcus erythropolis*	X5	0
GCA_009707305.1	*Rhodococcus sp. AQ5-07*	AQ5-07	0
GCA_009859915.1	*Rhodococcus sp. WAY2*	WAY2	1
GCA_009883715.1	*Rhodococcus rhodochrous*	ATCC BAA870	0
GCA_011611865.1	*Rhodococcus erythropolis*	KB1	0
GCA_012272955.1	*Rhodococcus sp. DMU1*	DMU1	0
GCA_013348805.1	*Rhodococcus sp. W8901*	W8901	1
GCA_014217765.1	*Rhodococcus triatomae*	DSM 44893	0
GCA_014217785.1	*Rhodococcus triatomae*	DSM 44892	1
GCA_014854695.1	*Rhodococcus rhodochrous*	BX2	1
GCA_015034605.1	*Rhodococcus qingshengii*	7B	0
GCA_015099595.1	*Rhodococcus qingshengii*	CS98	0

GCA_015169995.1	*Rhodococcus pyridinivorans*	5Ap	0
GCA_015654185.1	*Rhodococcus sp. M8*	M8	0
GCA_016025875.1	*Rhodococcus equi*	FDAARGOS_952	0
GCA_016598695.1	*Rhodococcus pyridinivorans*	B403	1
GCA_016722835.2	*Rhodococcus qingshengii*	F2-2	1
GCA_016804345.1	*Rhodococcus ruber*	C1	0
GCA_016839185.1	*Rhodococcus aetherivorans*	PSBB011	0
GCA_016879575.2	*Rhodococcus equi*	lh_12	0
GCA_017068035.1	*Rhodococcus erythropolis*	R85	0
GCA_017068375.1	*Rhodococcus koreensis*	R85	1
GCA_017068395.1	*Rhodococcus pseudokoreensis*	R79	1
GCA_017656075.1	*Rhodococcus sp. ZPP*	ZPP	1
GCA_017795725.1	*Rhodococcus qingshengii*	CX-1	0
GCA_017910955.1	*Rhodococcus qingshengii*	CL-05	0
GCA_018343855.1	*Rhodococcus sp. USK13*	USK13	1
GCA_019048925.1	*Rhodococcus qingshengii*	TG-1	0
GCA_019095205.1	*Rhodococcus pyridinivorans*	YC-JH2	0
GCA_019317345.1	*Rhodococcus sp. LW-XY12*	LW-XY12	0
GCA_019334125.1	*Rhodococcus globerulus*	D757	1
GCA_019378875.1	*Rhodococcus sp. USK10*	USK10	1
GCA_019703915.1	*Rhodococcus equi*	JCM94-14	0
GCA_019703935.1	*Rhodococcus equi*	JCM94-16	0
GCA_019703955.1	*Rhodococcus equi*	JCM94-25	0
GCA_019703975.1	*Rhodococcus equi*	JCM94-27	0
GCA_019703995.1	*Rhodococcus equi*	JCM94-31	0
GCA_019704015.1	*Rhodococcus equi*	JCM94-3	0
GCA_019704035.1	*Rhodococcus equi*	JID03-27	1
GCA_019704055.1	*Rhodococcus equi*	JID03-46	0
GCA_019704075.1	*Rhodococcus equi*	JID03-56	0
GCA_019856255.1	*Rhodococcus opacus*	DSM_44186	1
GCA_020542785.1	*Rhodococcus opacus*	PD630	1

GCA_020809065.1	*Rhodococcus sp. C1*	C1	0
GCA_020885655.1	*Rhodococcus sp. RDE2*	RDE2	1
GCA_021165915.1	*Rhodococcus aetherivorans*	CBO21-1	0
GCA_021165975.1	*Rhodococcus qingshengii*	VT6	0
GCA_021165995.1	*Rhodococcus pyridinivorans*	DNHP-S2	0
GCA_021441945.1	*Rhodococcus sp. DMF-1*	DMF-1	0
GCA_021497645.1	*Rhodococcus erythropolis*	D310-1	1
GCA_021654675.1	*Rhodococcus equi*	ATCC 33701	0
GCA_022059865.1	*Rhodococcus erythropolis*	CERE8	0
GCA_022179365.1	*Rhodococcus equi*	U19	0
GCA_022655485.1	*Rhodococcus opacus*	S8	0
GCA_022691665.1	*Rhodococcus equi*	P2120831	0
GCA_022691685.1	*Rhodococcus equi*	P2117036	0
GCA_022985635.1	*Rhodococcus opacus*	9	1
GCA_023100785.1	*Rhodococcus pyridinivorans*	YC-MTN	0
GCA_023221595.1	*Rhodococcus qingshengii*	djl-6	0
GCA_023238485.1	*Rhodococcus pyridinivorans*	PE41	0
GCA_023278385.1	*Rhodococcus ruber*	ZM15	0
GCA_023822085.1	*Rhodococcus pyridinivorans*	4-4	0
GCA_900478115.1	*Rhodococcus coprophilus*	NCTC10994	1

Supplementary Table S3. GenBank assembly numbers of the strains plus respective metadata used in this study. Isolation source abiotic / biotic refers to a host attached or free-living lifestyle of the strain

GENBANK ASSEMBLY ID	ISOLATION SOURCE	ISOLATION SOURCE_1	ISOLATION SOURCE_2	HABITAT/ LIFESTYLE	ISOLATION SOURCE_3
GCA_000010105.1	abiotic	water	water_marine	natural	pacific_ocean_water
GCA_000010805.1	abiotic	soil	soil_contaminated	contaminated	gasoline_contaminated_soil
GCA_000014565.1	abiotic	soil	soil_contaminated	contaminated	lindane_contaminated_soil
GCA_000196695.1	biotic	animal_host	horse	pathogenic	pneumonic_foal
GCA_000454045.1	abiotic	soil	soil_other	unknown	other_soil
GCA_000511305.1	abiotic	water	water_contaminated	contaminated	diesel_waste_site_water
GCA_000696675.2	abiotic	soil	rhizosphere	natural	rhizosphere
GCA_000760905.2	biotic	plant_host	flowering_plant	unknown	flowering_plant

GCA_000763325.2	abiotic	soil	soil_contaminated	contaminated	oil_polluted_soil
GCA_000954115.1	abiotic	water	water_marine	natural	arctic_sea_water
GCA_000975175.1	abiotic	soil	soil_plant_associated	natural	plant_associated
GCA_000982715.1	abiotic	soil	soil_contaminated	contaminated	contaminated_soil
GCA_001620005.1	biotic	plant_host	pistaccio_rootstock	pathogenic	pistaccio_rootstock
GCA_001620025.1	biotic	plant_host	pistaccio_rootstock	pathogenic	pistaccio_rootstock
GCA_001620305.1	biotic	plant_host	flowering_plant	unknown	Chrysanthemum
GCA_001682295.1	biotic	animal_host	ray	unknown	skate_renal_tissue
GCA_001685605.1	abiotic	sludge	activated_sludge	contaminated	contaminated_sludge
GCA_001700945.1	abiotic	soil	soil_contaminated	contaminated	PCB_contaminated_soil
GCA_001767395.1	biotic	animal_host	sponge	unknown	sponge_tissue
GCA_001886355.1	abiotic	soil	soil_other	unknown	other_soil
GCA_001942025.1	abiotic	soil	soil_other	unknown	other_soil
GCA_001984015.1	abiotic	soil	soil_plant_associated	natural	lawn_surface
GCA_002079265.1	abiotic	sludge	activated_sludge	contaminated	sludge_of_membrane_bioreactor_in_a_waste_water_treatment_plant
GCA_002094405.3	biotic	animal_host	horse	pathogenic	horse
GCA_002269365.1	abiotic	soil	soil_natural	natural	coastal_plane
GCA_002501585.1	abiotic	sediment	sediment_marine	natural	marine_sediment
GCA_002741725.1	abiotic	sediment	sediment_contaminated	contaminated	crude_oil_contaminated
GCA_002863905.1	abiotic	sludge	activated_sludge	contaminated	activated_sludge_from_pharmaceutical_waste_in_Shaoxing_China
GCA_002893965.1	abiotic	soil	soil_other	unknown	other_soil
GCA_003013675.1	abiotic	soil	soil_contaminated	contaminated	soil_near_a_pharmaceutical_factory_in_Beijing
GCA_003086595.1	abiotic	soil	soil_other	unknown	other_soil
GCA_003130705.1	abiotic	sediment	sediment_river	natural	river_sediment
GCA_003288095.1	abiotic	soil	soil_contaminated	contaminated	electronic_waste_recycling_area
GCA_003586525.1	abiotic	soil	soil_contaminated	contaminated	dyed_contaminated_soil
GCA_003641205.1	abiotic	soil	soil_rhizosphere	natural	rhizosphere
GCA_003860625.1	abiotic	water	antarctic_sea_ice	natural	antarctic_sea_ice
GCA_004006015.1	biotic	animal_host	mammal	unknown	tibetian_antilope
GCA_004328705.1	abiotic	sediment	sediment_freshwater	natural	freshwater_sediment
GCA_004795875.1	abiotic	soil	soil_natural	natural	Antarctica
GCA_004795915.1	abiotic	soil	soil_natural	natural	Antarctica
GCA_005484805.1	abiotic	air	air	natural	Singapore
GCA_005944105.1	abiotic	Contaminated site	site_contaminated	contaminated	Denitrifying_Bioreactor

GCA_006351985.2	abiotic	Contaminated site	water_contaminated	contaminated	Chilean_kraft_pulp_mill_effluents
GCA_007197835.1	biotic	human	human_lung_sputum	unknown	lung_sputum
GCA_007558985.1	abiotic	Contaminated site	site_contaminated	contaminated	Polycyclic_aromatic_hydrocarbons_contaminated
GCA_008306195.1	biotic	plant_host	plant_associated	unknown	rucola_leave_endophyte
GCA_008728655.1	abiotic	soil	soil_contaminated	contaminated	rubber_production_plant_territory
GCA_009707305.1	abiotic	soil	soil_natural	natural	polar_region
GCA_009859915.1	biotic	Bacterial consortium	bacterial_consortium	unknown	bacterial_consortium
GCA_009883715.1	abiotic	soil	soil_other	unknown	other_soil
GCA_011611865.1	abiotic	soil	soil_contaminated	contaminated	petroleum_contaminated_soil
GCA_012272955.1	abiotic	sludge	activated_sludge	contaminated	activated_sludge
GCA_013348805.1	biotic	human	blood	natural	blood
GCA_014217765.1	biotic	animal_host	insects	symbiotic	triatomine_bugs
GCA_014217785.1	biotic	animal_host	insects	symbiotic	triatomine_bugs
GCA_014854695.1	abiotic	Contaminated site	site_contaminated	contaminated	sewage_tank
GCA_015034605.1	abiotic	soil	soil_contaminated	contaminated	oil_refinery
GCA_015099595.1	abiotic	soil	soil_other	unknown	Japan
GCA_015169995.1	abiotic	soil	soil_contaminated	contaminated	Libyan_oil_polluted_soil
GCA_015654185.1	abiotic	soil	soil_contaminated	contaminated	nitrile_contaminated
GCA_016025875.1	biotic	human	patient	pathogenic	patient
GCA_016598695.1	abiotic	sludge	activated_sludge	contaminated	activated_sludge
GCA_016722835.2	abiotic	soil	soil_contaminated	contaminated	petroleum_contaminated_soil
GCA_016804345.1	abiotic	soil	soil_landfil_cover	contaminated	landfil_cover_soil
GCA_016839185.1	abiotic	Environmental sample	environmental_sample	natural	environmental_sample
GCA_016879575.2	abiotic	soil	soil_horse_breeding_farm	natural	horse_breeding_farms
GCA_017068035.1	abiotic	water	water_other	unknown	water_other
GCA_017068375.1	abiotic	soil	soil_rhizosphere	natural	rhizosphere
GCA_017068395.1	abiotic	soil	soil_rhizosphere	natural	rhizosphere
GCA_017656075.1	abiotic	soil	soil_other	unknown	other_soil
GCA_017795725.1	abiotic	soil	soil_contaminated	contaminated	carbendazim_contaminated_soil
GCA_017910955.1	abiotic	concrete	concrete	contaminated	concrete
GCA_018343855.1	abiotic	sediment	sediment_sand	natural	capable_of_degrading_Benzophenone_3
GCA_019048925.1	abiotic	water	water_contaminated	contaminated	oil_contaminated
GCA_019095205.1	abiotic	soil	soil_other	unknown	other_soil
GCA_019317345.1	abiotic	sludge	activated_sludge	contaminated	activated_sludge
GCA_019334125.1	abiotic	soil	soil_natural	natural	natural_resort
GCA_019378875.1	abiotic	sediment	sediment_river	natural	coastal_system_river_bank

GCA_019703915.1	biotic	human	patient	pathogenic	Aids_renal_transplant_lymphosarcom_ patients
GCA_019703935.1	biotic	human	patient	pathogenic	Aids_renal_transplant_lymphosarcom_ patients
GCA_019703955.1	biotic	human	patient	pathogenic	Aids_renal_transplant_lymphosarcom_ patients
GCA_019703975.1	biotic	human	patient	pathogenic	Aids_renal_transplant_lymphosarcom_ patients
GCA_019703995.1	biotic	human	patient	pathogenic	Aids_renal_transplant_lymphosarcom_ patients
GCA_019704015.1	biotic	human	patient	pathogenic	Aids_renal_transplant_lymphosarcom_ patients
GCA_019704035.1	biotic	human	patient	pathogenic	Aids_renal_transplant_lymphosarcom_ patients
GCA_019704055.1	biotic	human	patient	pathogenic	Aids_renal_transplant_lymphosarcom_ patients
GCA_019704075.1	biotic	human	patient	pathogenic	Aids_renal_transplant_lymphosarcom_ patients
GCA_019856255.1	abiotic	soil	soil_natural	natural	garden_soil
GCA_020542785.1	abiotic	soil	soil_contaminated	contaminated	gas_works_plant
GCA_020809065.1	abiotic	soil	soil_other	unknown	other_soil
GCA_020885655.1	abiotic	soil	soil_contaminated	contaminated	dumping_site_of_rubber_factory
GCA_021165915.1	abiotic	soil	soil_other	unknown	other_soil
GCA_021165975.1	abiotic	soil	soil_forest	natural	forest_soil
GCA_021165995.1	abiotic	soil	soil_other	unknown	other_soil
GCA_021441945.1	abiotic	water	water_marine	natural	marine_surface_water
GCA_021497645.1	abiotic	soil	soil_other	unknown	other_soil
GCA_021654675.1	biotic	animal_host	horse	pathogenic	foal_with_pneumonia
GCA_022059865.1	abiotic	soil	soil_contaminated	contaminated	active_remediatory_land_farm_site
GCA_022179365.1	biotic	animal_host	horse	pathogenic	foal_with_pneumonia
GCA_022655485.1	abiotic	soil	soil_forest	natural	forest_soil
GCA_022691665.1	biotic	animal_host	horse_feces	pathogenic	foal_feces_diseased_with_ pneumonia_and_diarrhea
GCA_022691685.1	biotic	animal_host	horse_feces	pathogenic	foal_feces_diseased_with_ pneumonia_and_diarrhea
GCA_022985635.1	abiotic	water	water_contaminated	contaminated	nitrophenolics_and_ trichloroethylene_contaminated ground_water
GCA_023100785.1	abiotic	sediment	sediment_river	contaminated	estuarine_mud_ (from_city_river_in_China)
GCA_023221595.1	abiotic	soil	sediment_sandy_loa m	natural	sandy_loam
GCA_023238485.1	abiotic	soil	soil_other	unknown	other_soil
GCA_023278385.1	abiotic	soil	soil_agricultural	natural	field_land

GCA_023822085.1	abiotic	soil	soil_other	unknown	other_soil
GCA_900478115.1	abiotic	sediment	sediment_lake_mud	natural	lake_mud

Supplementary References

1. Price MN, Dehal PS, Arkin AP. 2010. FastTree 2 – Approximately Maximum-Likelihood Trees for Large Alignments. PLoS One 5:e9490.

2. Letunic I, Bork P. 2021. Interactive Tree Of Life (iTOL) v5: an online tool for phylogenetic tree display and annotation. Nucleic Acids Res 49:W293–W296.
